# Role of Surface Bands in the Photogeneration, Cooling, and Recombination of Charge Carriers in Two-Dimensional Bi_2_Se_3_

**DOI:** 10.1021/acsnano.4c14134

**Published:** 2025-05-05

**Authors:** Jara F. Vliem, Servet Ataberk Cayan, Riccardo Reho, Andrés R. Botello-Méndez, Pieter Geiregat, Zeila Zanolli, Daniel Vanmaekelbergh

**Affiliations:** † Debye Institute for Nanomaterials Science, Utrecht University, Princetonplein 1, Utrecht 3584, The Netherlands; ‡ Physics and Chemistry of Nanostructures, Ghent University, Gent 9000, Belgium; § NoLIMITS Center for Non-Linear Microscopy and Spectroscopy, Ghent University, Gent 9000, Belgium

**Keywords:** ultrafast pump–probe spectroscopy, topological insulator, colloidal nanoplatelets, surface states, first-principles DFT

## Abstract

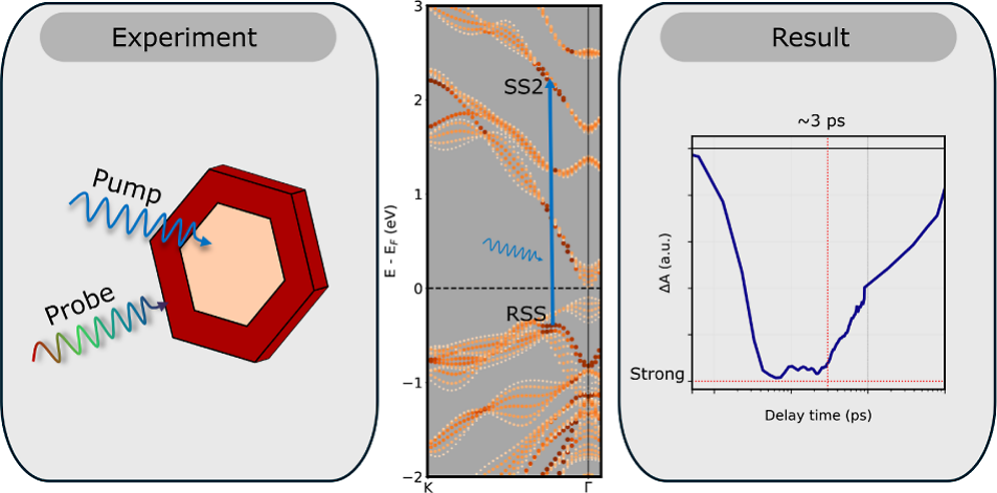

Bi_2_Se_3_, a layered three-dimensional topological insulator, exhibits intriguing changes in its band structure when its thickness is reduced below 7 quintuple layers. The reduction in thickness leads to hybridization between the surface states and the opening of a gap between these states. We combine density functional theory calculations with pump–probe spectroscopy to explore how these hybridized states affect the photogeneration, cooling, and recombination of charge carriers in two-dimensional Bi_2_Se_3_ nanoplatelets. Our calculations reveal that the hybridized surface states are crucial for understanding the optical transitions. By comparing the experimental absorption spectrum with the calculated absorptance in the near-infrared-visible region, we identify key transitions within the 2D Brillouin zone. We distinguish transitions involving the hybridized surface states from those involving the interior layers. We observe a significant delay of several picoseconds in carrier recombination when surface state transitions are excited, which we attribute to carrier accumulation in the valleys of the Rashba-shaped surface-state valence band and in higher-lying surface states of the conduction band. These findings emphasize the important role of surface state bands in the optical behavior of Bi_2_Se_3_ and their potential for manipulating carrier dynamics in two-dimensional materials.

## Introduction

Bismuth selenide (Bi_2_Se_3_), a layered van der Waals material composed of quintuple layers (QLs, i.e., Se–Bi–Se–Bi–Se layers), has been the subject of intense research since bismuth chalcogenides were found to be among the best thermoelectric materials.^1−4^ More recently, Bi_2_Se_3_ has gained interest due to its topological properties: bulk Bi_2_Se_3_ is a well-known three-dimensional (3D) topological insulator in which the inverted gap of 200–300 meV hosts a helical Dirac-cone surface state characterized by spin-momentum locking.^5−7^ Besides the electronic topology that depends on the band structure around the Fermi level, the physics of optical excitations involving bands considerably below and above the Fermi level is also of interest.^8−10^ Recently, Kung et al. (2019)^11^ demonstrated that the surface states of 3D Bi_2_Se_3_ play an important role in optical transitions in the visible region, resulting in circularly polarized emission at 2.3 eV. This emission was attributed to the recombination of chiral excitons, composed of electrons with zero effective mass and massive holes on the material’s surface. However, when the thickness of 3D Bi_2_Se_3_ is reduced below 7QLs, ARPES measurements have shown that the surface states involved in these transitions hybridize and become gapped.^6,12,13^ Such hybridization of states in topological insulators, along with topological phase transitions from 3D to 2D nontrivial materials, can result in exotic physics.^14−18^ Existing reports have indicated that the gapped surface states may still significantly affect the (optical) properties of 2D Bi_2_Se_3_,^19−22^ but how these surface state bands (versus interior bands) determine the optical properties of 2D Bi_2_Se_3_ in the near-infrared-visible (NIR-VIS) has not been investigated systematically. Here, this question is addressed by combining density functional theory (DFT) calculations with ultrafast pump–probe spectroscopy on colloidal nanoplatelets (NPLs) of approximately 6QLs thick and with lateral dimensions in the 200 nm range. We focused on platelets of this thickness as our work shows that they can be reproducibly synthesized and are chemically and mechanically robust. Furthermore, these free-standing crystals have a uniform and controlled thickness, and their lateral dimensions are large enough to classify them as genuinely two-dimensional.^13^ Our DFT calculations on 6QL slabs of Bi_2_Se_3_ show that the states associated with the gapless surface states of 3D Bi_2_Se_3_ become an integral part of the 2D band structure by forming regions with a high surface contribution in the bands. By comparing the experimental absorbance with the theoretical absorptance, we identified the most important transitions in the 2D Brillouin Zone (BZ) and located them in the calculated band structure. Our analysis revealed three primary groups of transitions: on the line Γ-K close to Γ involving the gapped surface state bands, around Γ involving interior QLs, and another group involving the interior QLs, but containing transitions more broadly distributed over the BZ. We combined these theoretical findings with our pump–probe experiments to show that delayed recombination occurs whenever bands with a large surface state contribution are involved. Specifically, our results point to the accumulation of holes at the valleys of a Rashba-like Surface State (RSS)^11^ band, and the accumulation of electrons in higher lying surface states of the conduction bands. This explains the above-optical-bandgap photoluminescence observed in the literature.

## Results and Discussion

**Figure 1:**
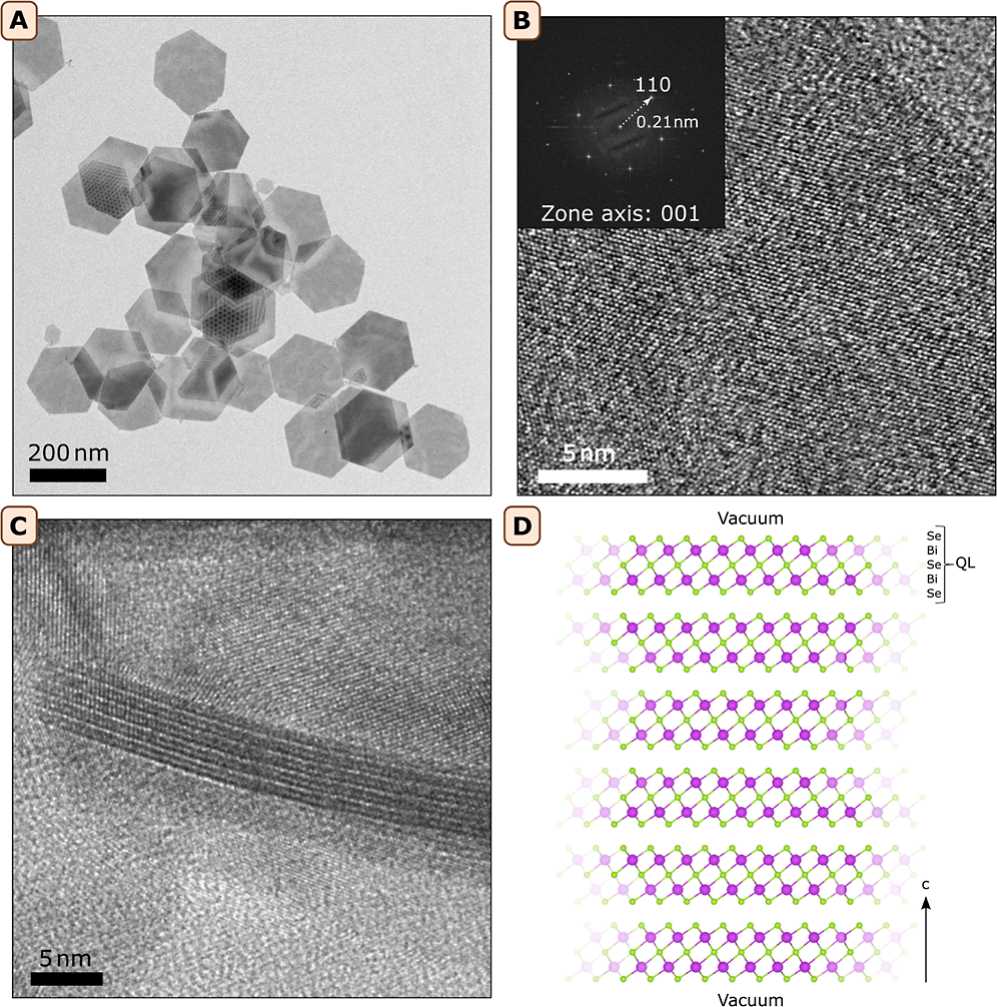
Synthesized Bi_2_Se_3_ NPLs. (A) TEM image of representative NPL sample. (B) High resolution TEM image of a NPL edge, with a FFT inset to show the crystallinity of the platelet. (C) TEM image of a 5QL NPL from the side. The Bi atoms can be recognized as darker lines in the layered structure. (D) Atomistic model of 6QLs Bi_2_Se_3_ employed in the theoretical calculations. The structure has infinite lateral dimensions and the stacking direction is along the *c*-axis.

**Figure 2:**
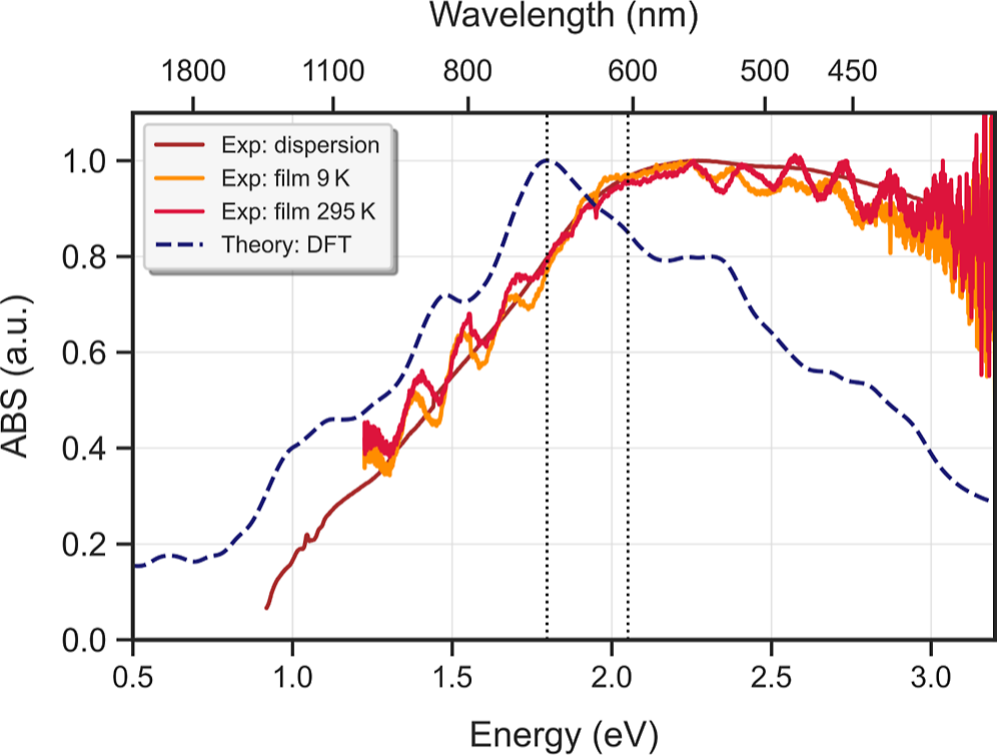
Comparison between the theoretical absorptance and experimental absorption spectra. Absorption spectra of Bi_2_Se_3_ NPLs in dispersion and drop-cast on films (at 295 and 9 K), and the DFT absorptance for an infinite Bi_2_Se_3_ slab of 6QLs. The dashed lines indicate the 0.25 eV shift between the experimental and theoretical spectra.

The optical measurements described in this work were conducted on dispersions and drop-cast films of Bi_2_Se_3_ NPLs (Figures 1 and S1). The synthesis procedure is outlined in the experimental section. Figure 1A shows a representative TEM image of the prepared hexagonal platelets. In Figure 1B, a high-resolution TEM image of a nanoplatelet is shown with the corresponding Fast Fourier Transform (FFT), showing the crystallinity and orientation of the nanoplatelet. Figure 1C shows a nanoplatelet of 5QLs imaged from the side in which the bismuth atom columns (see Figure 1D) can be seen as darker lines. The prepared NPLs have a lateral size of 180 ± 61 nm (TEM, 280 NPLs) and an average thickness of 6QLs (Supporting Information, Figure S2). In order to relate the optical properties of the NPLs to their electronic band structure, we compared experimental and theoretical absorption spectra in Figure 2. The experimental spectra were measured on both suspensions and films, with the films measured at 295 and 9 K. The spectra cover the NIR-VIS region, well above the fundamental (inverted) gap. The absorbance of the film and dispersion are nearly indistinguishable, with the only noticeable difference being the Fabry–Pérot oscillations in the film spectra. From 1 to 2.2 eV, the spectra show a strong increase in absorbance, which then gradually decreases up to 3.0 eV. This is qualitatively similar to the absorbance spectra reported for 3D Bi_2_Se_3_ crystals.^23,24^ The theoretical absorptance, shown in the same figure, was computed using first-principles DFT. Because the NPLs are on average 6QLs thick, the calculations were performed for infinite slabs of Bi_2_Se_3_ with a thickness of 6QLs (Figure 1D). Here, we have made use of the independent particle (IP) approximation, which yields results similar to those of the more expensive GW-BSE calculation. This approach is justified as our analysis revealed that the main differences when using many-body approaches include a slight relative increase in absorptance at lower energies and a minor red-shift of approximately ∼100 meV (see Supporting Information, Figure S3). A detailed explanation is given in the computational details section. All calculated transitions were artificially broadened by 10  meV to account for both homogeneous and inhomogeneous broadening effects in the experimental absorbance, resulting in a good qualitative agreement with the experimental spectrum. We expect the inhomogeneous broadening to be largely due to variations in the thickness of the NPLs, which ranges from 5 to 7QLs.^23^ An inhomogeneous spatial distribution of the charged polymer capping around the platelets could additionally induce a spatially inhomogeneous Stark effect, mostly resulting in broadening. However, we remark that our experimental absorption spectrum is very similar to that of vapor–solid-deposition (VSD)-grown samples, or to samples prepared with different surfactants, showing that the influence of surfactants in our synthesis is minimal.^25,26^ Although the overall shape of our computed absorptance is in good agreement with the experimental absorption, we observe two main discrepancies. First, we find a red-shift of approximately 0.25 eV relative to the experimental spectrum. Such a shift is often observed between theory and experiment, as outlined in the computational details, and we will correct for this in the remainder of our argumentation. Second, in the higher-energy region above 2.5 eV, the experimental absorbance is stronger than the calculated absorptance. The absorbance in this region has previously been attributed to excitations into higher-lying electronic bands that are not included in our model, which may account for the observed difference.^23^ Since our analysis focuses on transitions occurring between 1 and 3 eV, absolute agreement between theory and experiment in the higher energy region is not critical to our argumentation.

**Figure 3:**
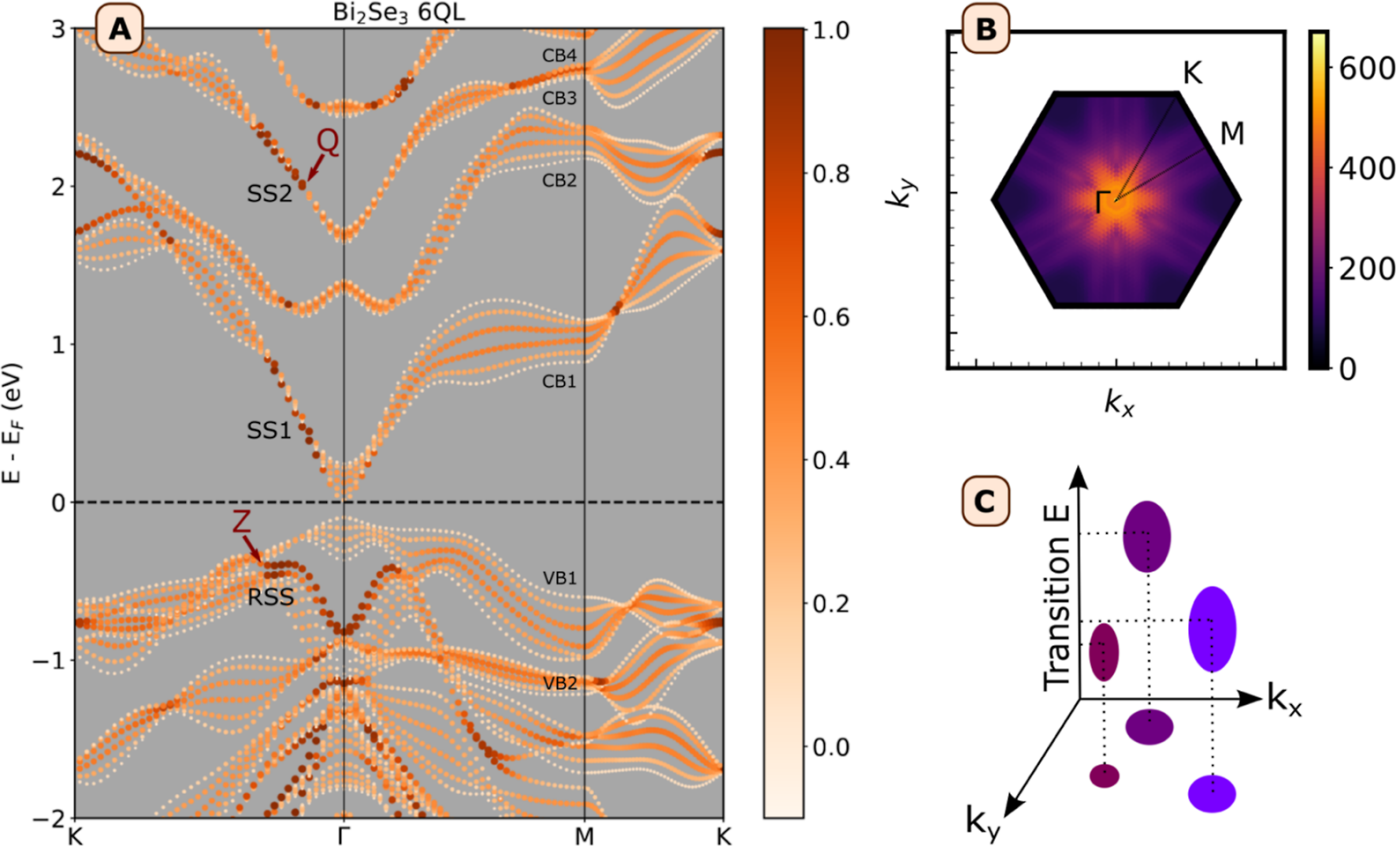
Band structure and k-resolved absorptance map of 6QL Bi_2_Se_3_. (A) Electronic band structure for 6QL Bi_2_Se_3_, with surface layer contributions projected onto the band structure. The color scale indicates the surface layer contribution (scale 0–1). The conduction and valence band manifolds have been numbered for clarity and the surface state bands RSS, SS1, and SS2, are indicated. Z indicates the end of the RSS bands on the line Γ-K and Q is the charge transfer point at the bottom of SS2. (B) Map of absorptance resolved by wavevector *k*, with the integrated absorptance from 0.5 to 3.0 eV displayed in arbitrary units. The absorptance is projected onto the (*E* = 0, *k*_*x*_, *k*_*y*_) plane, representing the 2D BZ. (C) Diagram showing how the k-resolved absorptance maps are constructed by projecting the transitions onto the BZ.

Now that we have established satisfactory agreement between theory and experiment, we will proceed to use our theoretical model to understand the origin of specific strong transitions in 2D Bi_2_Se_3_. After this, we will discuss our pump–probe experiments to explore, using our theoretical understanding, how the hybridized surface states of 2D Bi_2_Se_3_ influence its optical properties. In Figure 3A, we show the band structure computed for infinite slabs of 6QL Bi_2_Se_3_ by using the IP approximation. The contribution of the outermost QLs (surface layers) to the states is represented with darker and thicker symbols, highlighting regions in the band structure characterized by significant contributions from the surface QLs. We verified that the states in dark red are indeed located at the surface by visualizing the contribution to the charge density (Figure S4). Therefore, these regions in the band structure correspond to the dispersive surface states discussed in the literature, and we will refer to them as such.^11,27^ We followed the convention of Kung et al.^11^ to label the surface state bands: we identified the RSS band and the empty first and second surface state bands (SS1 and SS2) forming part of the conduction band manifolds CB1 and CB3 respectively, similar to those found in bulk Bi_2_Se_3_ (supporting Figure S5). In the 2D case discussed here, the states from the surface and interior layers become mixed. Due to this mixing, there are several points of interest in the calculated band structure. We have indicated two points that will be useful for our later explanations: Z and Q. Z indicates the end of the RSS bands on the line Γ-K, and Q is the point at the bottom of SS2 where further relaxation toward Γ in CB3 involves charge transfer from surface to interior layers. We will, therefore, call this the charge transfer point.

By combining the computed band structure with the computed absorptance, we can uniquely assign each optical transition to a specific momentum and energy, effectively mapping them within the band structure. For this analysis, we present the absorptance data in an energy, momentum, and absorbance frame directly related to the band structure. In this way, we create a unique mapping between optical transitions and their origin electronic states (transitions from valence to conduction bands), with the final goal of distinguishing surface from bulk transitions via the weighting factor of the interior orbitals. An example of these (energy, momentum, absorbance) maps is shown in Figure 3B. Figure 3C shows how to interpret such k-resolved absorptance maps: in a selected energy range, the strength of the optical transitions is calculated and subsequently projected onto the 2D BZ with a mesh of 2304 projection points. For example, in Figure 3B the false-color plot shows the absorptance strength integrated over the entire energy window of 0.5–3.0 eV. The color scale indicates the strength of the transitions (i.e., integrated absorptance) in arbitrary units. From this k-resolved absorptance map, we find that the strongest transitions occur at Γ and on the line Γ-K. We note that when transitions starting from *E* = 0 are included, the integrated value at Γ is so large that it occludes the other transitions (supporting Figure S6).

**Figure 4:**
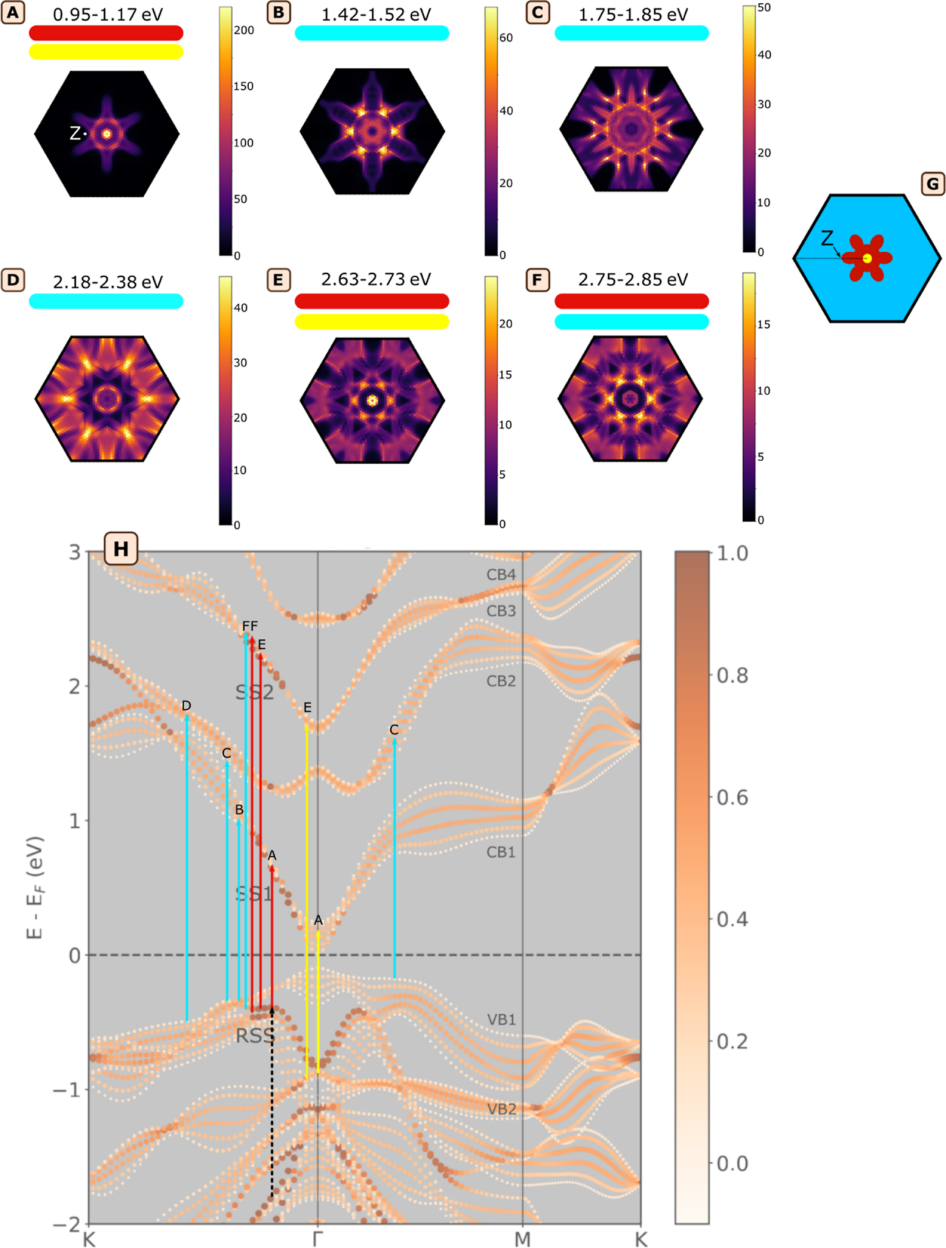
Identified transitions in the band structure of 6QL Bi_2_Se_3_. (A–F) k-resolved absorptance maps integrated over 0.95–1.17 , 1.42–1.52, 1.75–1.85, 2.18–2.38, 2.63–2.73, and 2.75–2.85 eV. The color scale shows the integrated absorptance (a.u.). The colored bars underneath the energy range indicate to which color group the transitions belong to as indicated in panel *G*. (G) Schematic of the BZ separated into color zones: transitions close to Γ belong to yellow, Γ-K involving RSS to red, and other transitions involving interior QLs to blue. Z indicates the end of the RSS bands on the line Γ-K. As a visual aid, a *Z* point is also shown in panel *A*. (H) Band structure for 6QL Bi_2_Se_3_ with located transitions A–F. The energy of these transitions correspond to the mean of each energy range in panels A–F. Their color corresponds to the group they belong to (see panel *G*). The dashed black arrow indicates a possible photoinduced absorption (PA) involving surface bands.

**Figure 5:**
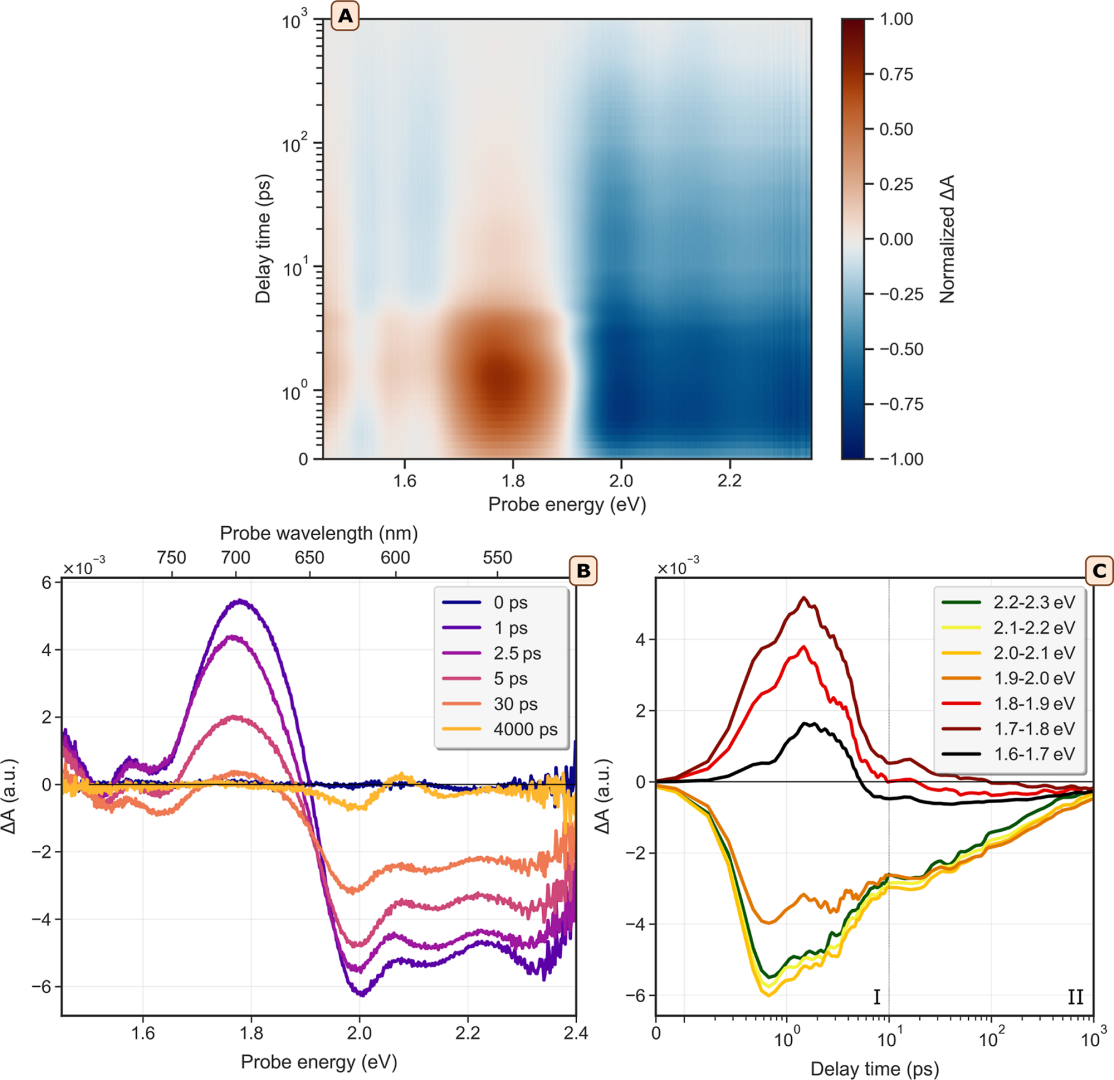
Time resolved pump (2.82 eV)-probe spectroscopy on a film of Bi_2_Se_3_ nanoplatelets. (A) Transient absorption map showing the normalized Δ*A* of a drop-cast Bi_2_Se_3_ NPL film at 15 K, after photoexcitation at 2.82 eV (440 nm). (B) The transient bleach spectrum at various pump–probe delay times. A pump power of 426 μW was used, which translates to a fluence of 4.2 × 10^13^ photons/cm^2^. (C) Traces of Δ*A* as a function of pump–probe delay time. The data are averaged over each 0.1 eV between probe energies of 1.6–2.3 eV to reduce noise. The dotted line around 10 ps divides the figure into regime I and II, which show different kinetics.

To obtain the location of specific strong transitions, we selected small energy windows containing strong transitions of the calculated absorptance spectrum (supporting Figure S7). The k-resolved absorptance maps integrated over these selected energy ranges are shown in Figure 4A–F. For each energy range, the strongest transitions occur in specific regions in the BZ. Based on their position, we found it instructive to group the transitions into three color categories as indicated in Figure 4G: transitions at or close to Γ involving interior QLs (yellow), transitions on the Γ-K line involving the top of RSS (red), which we refer to as surface state transitions due to the larger contribution of the surface layers, and last transitions more broadly distributed over the BZ involving interior QLs (blue). We will use these colors to refer to groups of transitions in the remainder of this text. The colors of the bars in Figure 4A–F indicate which groups are predominantly involved in a given energy range. For instance, in panel *A* (*E* = 0.95–1.17 eV), the strongest transitions occur at Γ (yellow group) and on line Γ-K (red group). We show the positions of these transitions in the band diagram (see yellow and red arrows denoted A). Similarly, for panel *D* (2.18–2.38 eV), we can identify strong transitions halfway along the line Γ-K. These transitions belong to the blue group, as they originate from a position closer to K than the end of the RSS band, i.e., to the left of point Z in Figure 4G. Since the transitions at this energy are more distributed across the BZ, arrow D in the band diagram represents only one possible position along the line Z-K. There are additional transitions around the path M-K, but considering their wide distribution in *k*-space, we do not show them in the band structure. Analogously, for panel *E* (*E* = 2.63–2.73 eV), we identified only the strongest transitions in the band diagram belonging to the yellow and red groups. This procedure was repeated for the other strong transitions within the energy range of 0.5–3.0 eV, where we have primarily considered transitions from the top of VB1 (at Γ), the RSS band, or the top of VB2 (at Γ). Notably, the transition in panel *F* on the line Γ-K can be positioned both at the top of RSS involving surface states (red group, right of Z), or just next to it left of Z, making it part of the blue group. As shown in the band diagram of Figure 4H, a significant part of the identified strong transitions involves the hybridized surface bands. Using this knowledge, we now proceed to study how these bands affect the cooling and recombination dynamics in our 2D Bi_2_Se_3_ NPLs. We performed ultrafast transient absorption spectroscopy with a broad range of pump energies ranging from 1.18 to 3.6 eV to probe the higher-lying excitations (see Experimental section). A 170 fs pump pulse was used to excite a drop-cast film of Bi_2_Se_3_ NPLs (Figure 1), followed by a broadband pulse to probe the sample after a time delay *t*. This gives the change in absorbance, Δ*A* = *A*–*A*_0_, where *A*_0_ is the absorbance of the sample without prior excitation by the pump. We first discuss the data obtained with a pump energy of 2.82 eV in detail, which is shown in Figure 5.

Figure 5A presents a 2D map of the differential absorbance Δ*A*(λ, *t*) measured at 15 K for excitation at 2.82 eV (440 nm). We used low-temperature data acquired on films to achieve the best-resolved signals, although measurements at room temperature and in dispersion yield similar results (Supporting Information, Figures S8 and S9). The 2D map in Figure 5A displays two different responses: a bleach signal (Δ*A* < 0) at probe energies above 1.9 eV and a photoinduced absorbance (PA, Δ*A* > 0) at energies below 1.9 eV, in line with the literature.^20^ These features are more easily distinguishable in panel *B*, which shows time-resolved traces with distinct bands around 2.0, 2.15, and 2.30 eV (bleach), and 1.8, 1.6, and 1.45 eV (PA). These bands could partly originate from Fabry–Pérot oscillations in the film spectra, as indicated by the similarity between the fringe spacing in the linear absorption spectrum and that observed in the transient absorption data. In panel *C*, the time evolution of the bleach and PA signal at selected probe energies is shown, averaged over each 0.1 eV of the probe range to improve the signal-to-noise ratio. PA dominates at lower energies, while bleach is more prominent at higher probe energies. The maximum bleach occurs around probe energies of 2.0–2.1 eV, coinciding with the maximum in the linear absorption spectrum. The plot can be divided into two regimes, I and II, where the kinetics differ significantly. In regime I, the decay of both PA and bleach is different from regime II, where the decay exhibits a mixture of first and second order kinetics^28^ (supporting Figures S14 and S15). For recombination at Γ, we expect that photogenerated (cooled) holes recombine with donor-type electrons^6,8,13,29^ (expected first order kinetics), while in other regions photogenerated electrons and holes recombine (expected second order kinetics). However, a complete kinetic interpretation is complex, as there are several regions in the 2D BZ where recombination can occur.

**Figure 6:**
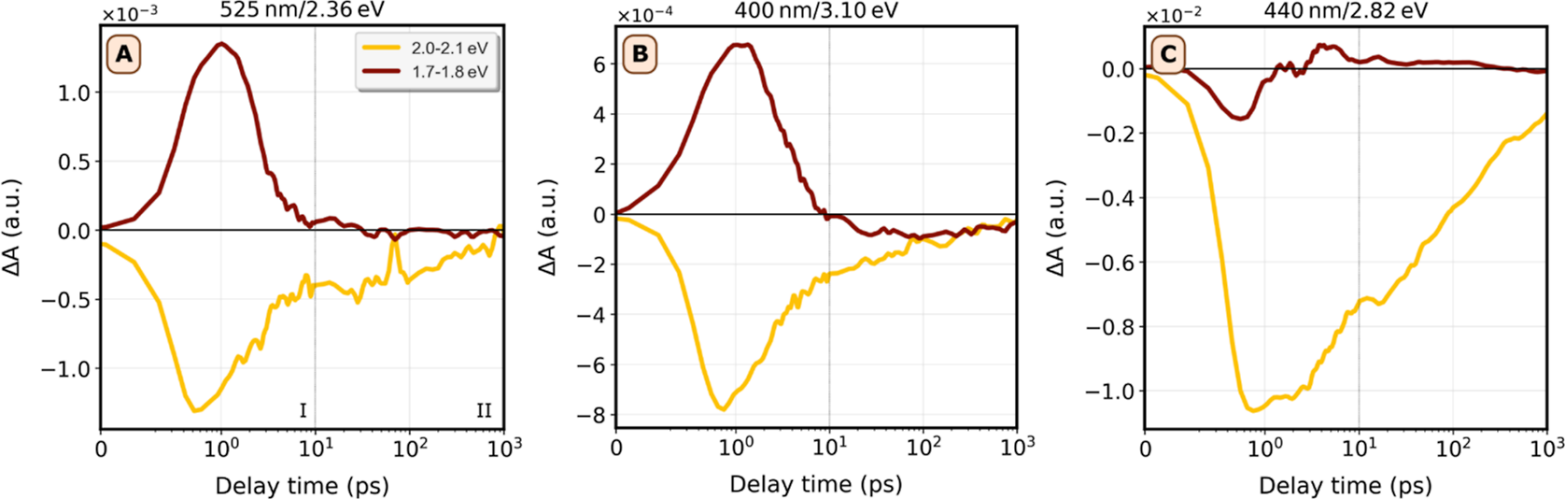
Decay kinetics of a film of Bi_2_Se_3_ nanoplatelets at selected pump energies. Traces of Δ*A* as a function of pump–probe delay time for a drop-cast Bi_2_Se_3_ NPL film measured at 15 K, after photoexcitation at (A) 2.36, (B) 3.10, and (C) 2.82 eV. The data are averaged over 0.1 eV between probe energies of 1.7–1.8  and 2.0–2.1 eV to reduce noise. The dotted lines around 10 ps divide the figures into regime I and II, which show different kinetics. A fluence of 8 × 10^13^ photons/cm^2^ was used for all measurements. Note: this is 2*x* higher than the fluence used in Figure 5.

We will now use our theoretical understanding to explain the bleach and PA. The supporting schematic in Figure S10A may be used for the sake of clarity. First, we examine using our theoretical band structure what can occur upon excitation with an experimental excitation energy of 2.82 eV. This energy corresponds to a theoretical excitation of (2.82–0.25) = 2.57 eV, which results in electrons generated in CB3 (transition E in Figure 4) and CB4 (VB1 → CB4 at Γ, not indicated). These electrons may relax to the lower conduction bands, specifically to the valleys between Γ and K in CB2, thereby bleaching several transitions around Γ. Additionally, holes are generated at the top of the RSS and in the interior layers at Γ (VB1 and VB2). The latter may cool to the valleys in VB1 and RSS, where they can bleach the blue and red groups of the transitions. Returning to our experimental data, we find that the region of strong bleach, identified above as ranging from 2.0 to 2.3 eV, corresponds to a theoretical region of 1.75–2.05 eV. The k-resolved absorptance plots in Figures 4 and S12 show that these energies correspond to transitions predominantly in the blue group (i.e., Figure 4C,D). Thus, we conclude that the bleach is largely caused by accumulation of holes in the local maximum of VB1 that bleach the blue group of interior state transitions. The PA is more challenging to assign, as these transitions are absent in the ground state (linear) absorption. However, for the holes generated at Γ (interior layers), we remark that there is nearly a continuum of states below VB1, enabling the photoexcitation of these holes roughly between 1 and 2 eV. Similarly, for the holes that are generated or accumulate in the RSS valley between Γ-K, several surface band photoexcitations are possible. We tentatively ascribe the broad PA centered around 1.8 eV to the photoexcitation around Γ, and the PA around 1.45–1.6 eV to the photoexcitation from RSS to the lower-lying surface states (dashed black arrow in Figure 4H). Our assignment does not exclude photoinduced excitations of conduction band electrons into the higher-lying conduction bands, nor can we entirely exclude the contribution of bandgap renormalization (BGR) to the PA signal (with an estimated maximum of 5%, see Supporting Information Figure S11 for a detailed explanation). To specifically investigate the role of surface state bands, we performed multiple pump–probe experiments at different pump energies to selectively excite bulk or surface state bands. The data, obtained with comparable fluence and presented as energy-resolved traces, are shown in Figure 6, for pump energies of 3.10, 2.82, and 2.36 eV. The data are again averaged over 0.1 eV between probe energies of 1.7–1.8 and 2.0–2.1 eV. The full data set for probe energies between 1.6 and 2.3 eV is shown in the Supporting Information, Figure S13. Time-resolved traces for these measurements presented in Figure S16, and additional measurements at other pump energies can be found in the Supporting Information (figures S17 for films and S18 for dispersions). First, we remark that Figure 6A and B are comparable in both the magnitude of the bleach and PA, although the PA at lower pump energy (2.36 eV) is more pronounced relative to the bleach signal. The traces also display qualitatively similar behavior: both PA and bleach exhibit a maximum around 1 ps, followed by decay up to 10 ps (i.e., regime I). After 10 ps, the slope changes (regime II). In contrast to A and B, the measurement in panel *C* at a pump energy of 2.82 eV exhibits qualitatively different behavior. First of all, the magnitude of the bleach is considerably larger and hardly any PA is observed relative to the bleach. Second, the maximum bleach for probe 2.0–2.1 eV remains approximately constant for about 5 ps before decaying. Notably, we observe such a delayed recombination predominantly for the range of pump energies from 2.7 to 3.0 eV, as shown in Supporting Information, Figure S18.

We will again use our theoretical model to explain these observations. Taking into account the energy difference between theory and experiment, we relate the experimental excitation in the energy region between 2.7 to 3.0 eV to a theoretical excitation of 2.45–2.75 eV. From Figure S19, we find that k-resolved absorptance plots for this energy range all show relatively strong transitions from k-regions around Γ and the top of the RSS band between Γ and K, suggesting that these bands might be involved. From the band diagram, we find that the RSS → SS2 transitions occur between *E* = 2.35–2.75 eV, thus closely matching the transition energy for which we observe a constant and strong bleach signal. We therefore attribute the delayed recombination mainly to the involvement of the RSS and SS2 bands (i.e., surface state transitions). To corroborate our argument, we refer again to Figure S19. Above 2.9 eV, we find that the RSS bands are not involved in the dominating transitions (i.e., most transitions occur to the right of point Z along the line Γ-K), while below 2.35 eV, our band diagram shows that the RSS-SS2 transition is energetically not possible.

Since we established that the observed strong bleach and delayed recombination in time regime I are related to specific pump energies that involve the RSS → SS2 transition, we can attempt to explain the reason for this using our calculated band structure. An illustration of our reasoning is also shown in Figure S10B. Upon excitation, transitions may occur around Γ and from RSS → SS2. Transitions from RSS → SS2 result in holes in the RSS valley and in VB1 between Γ-K, which temporarily accumulate there. Regarding the electrons photogenerated in SS2, these may cool toward the Γ point until they reach the charge transfer point Q between interior and surface layers indicated in Figure 3A. At this point, the transfer of charge from the surface to the inner QLs could create a bottleneck, hindering further cooling. Considering the geminate recombination of electrons and holes, these effects might result in an accumulation and temporary separation of photogenerated charge carriers in momentum space, causing a delayed electron–hole recombination at Γ. This also holds for electrons photogenerated at Γ, considering that holes may cool to the top of RSS or VB1. Hence, we ascribe the experimentally observed constant bleach in time regime I to the remarkable shape of the RSS band and the SS2 surface state band. We note that similar accumulation effects have been reported for bulk^8^ or MBE grown Bi_2_Se_3_.^20^ When instead of RSS → SS2 transitions, transitions between interior layers are pumped (e.g., using pump = 3.1 or 2.36 eV), charge carriers can more rapidly cool to the Γ point where they can recombine, and no delayed recombination is observed.

We tentatively explain the difference in the magnitude of the bleach between various pump energies with the distribution of charge carriers over the BZ. When exciting from RSS to SS2, electrons may accumulate temporarily at the charge transfer point in the SS2 bands while holes can relax to the valleys of VB1 and RSS. In addition, holes that have been photogenerated at Γ at these excitation energies (i.e., transition E in Figure 4H) may also relax to the valleys of VB1. As such, the redistribution of carriers in the BZ separates electrons and holes in momentum space. The holes in RSS quench the overall strong absorption on the line Γ-K (red group), while holes that have accumulated in the valleys of VB1 may also quench part of the blue group. Hence, both red (surface) and blue (bulk) group transitions are simultaneously affected and the bleach signal is amplified. In contrast, when using excitation energies in the blue group (e.g., 2.36 and 3.1 eV) carriers are not necessarily separated in momentum space and may recombine faster, resulting in the quenching of fewer transitions and thus a smaller bleach signal.

**Figure 7:**
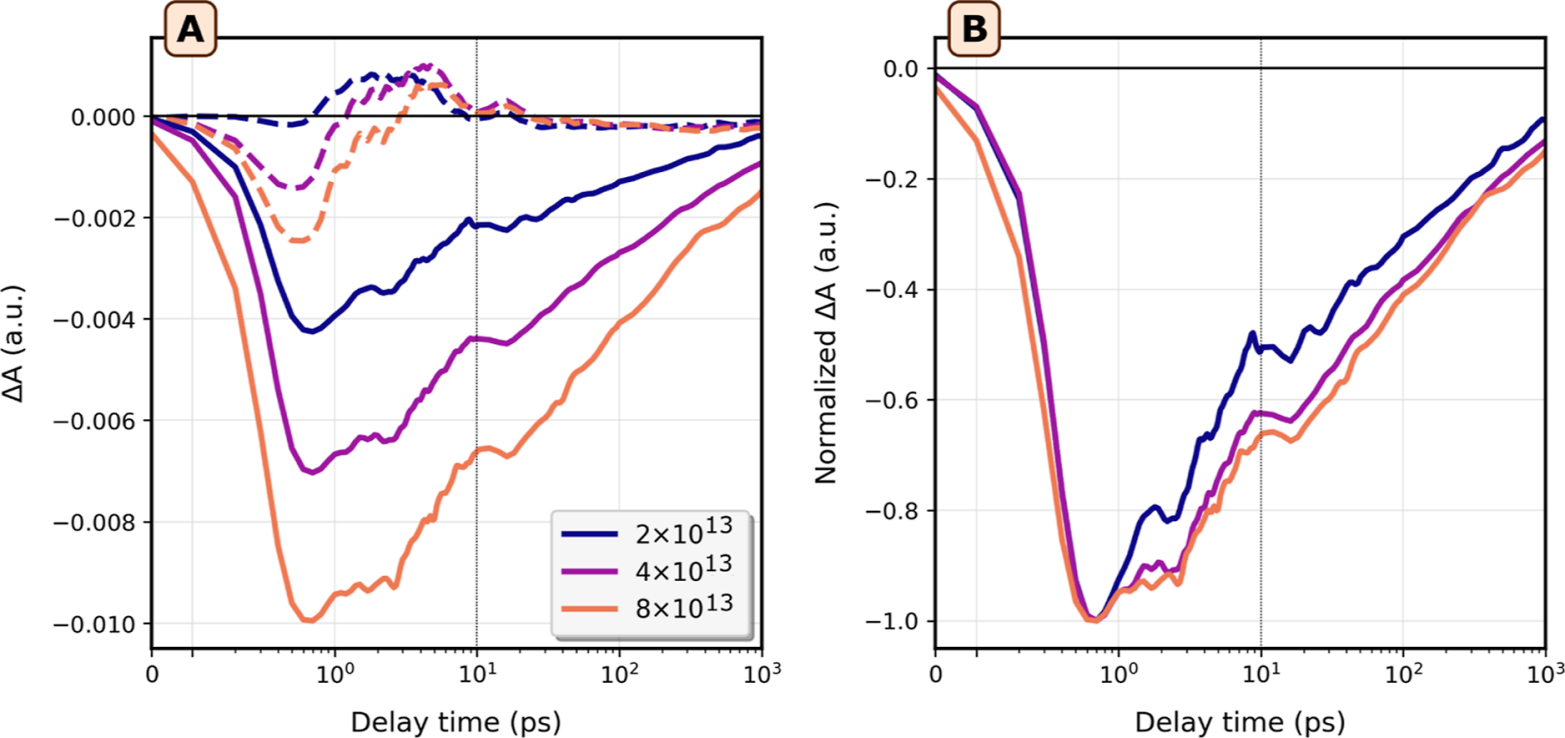
Cryogenic pump (2.82 eV)-probe spectroscopy on a film of Bi_2_Se_3_ nanoplatelets at various fluences. (A) Traces of Δ*A* against delay time of a dropcast Bi_2_Se_3_ NPL film at 15 K, after photoexcitation at 2.82 eV. Dashed traces are averaged between probe energies of 1.6 and 1.7 eV, and solid traces are averaged between probe energies of 1.9 and 2.0 eV. The fluence used for each experiment is given in the legend, in photons/cm^2^ (B) normalized traces of (A) averaged for probe energies between 1.9 and 2.0 eV.

Now that we understand the origin of the delayed recombination, we will finally discuss the fluence dependence of the bleach kinetics, illustrated in Figure 7. Figure 7B shows that a significant delay of ±5 ps in the bleach can be observed during regime I that becomes more prominent at higher fluences, while the kinetics in regime II does not change. Additional figures showing the fluence dependence for various excitation energies are provided in the Supporting Information and Figure S20. From this figure, it is evident that for pump energies where RSS → SS2 transitions are not excited, delayed recombination only becomes observable at extremely high fluences (see also Figure S21). This further shows that the recombination delay observed when pumping between 2.7 and 3.0 eV is strongly correlated with the accumulation of holes and electrons, which in this case likely occurs in the valleys of RSS/VB1 and at the charge transfer point in SS2, respectively. We remark here that we checked, using the linear absorption spectrum, that the samples were not degraded after the experiments. When the samples are irradiated to the point of damage, the recombination kinetics become considerably faster (Supporting Information, Figure S22). We will end our discussion by comparing our results with literature reports. We find that our conclusions align with those of Kung et al.^11^ among others,^19,21,30^ underscoring the significant contribution of surface states to the optical properties of Bi_2_Se_3_. Specifically, Kung et al. observed a photoluminescence (PL) peak centered around 2.3 eV with preserved polarization when exciting a 3D Bi_2_Se_3_ crystal between 2.6 and 3.0 eV, alongside a nonpolarized PL peak at 1.5 eV. The highest PL intensity of the polarized PL peak occurred at an excitation energy of 2.8 eV. As mentioned before, we find from the band diagram that RSS-SS2 transitions can occur between *E* = 2.35–2.75 eV, corresponding to an experimental excitation energy of *E* = 2.60–3.00 eV. This range matches the range for which polarization preserving PL was observed in ref (11). Following excitation at these energies, carrier accumulation at the charge-transfer point Q (Figure 3A) may enhance radiative transitions back to the valleys of RSS or VB1 (2.3–2.4 eV, including the experimental shift), thereby accounting for the observed luminescence at 2.3 eV. Please note that the emission at 2.3 eV from a material with a bandgap of ∼0.3 eV is highly remarkable in its own regard. The observed PL at 1.5 eV may result from electron relaxation to SS1, after which electrons and holes can recombine at the top of RSS. These findings highlight several distinct optical properties of layered Bi_2_Se_3_ compared to traditional semiconductors: (a) transitions involving the outer QLs (surface state transitions) form an important contribution to the overall optical properties; (b) high-energy excitations do not necessarily decay to the primary band gap but can be emitted as high-energy photons; (c) electrons and holes may separate in momentum space by cooling in anomalously shaped bands; and (d) the bands have charge-transfer and saddle points where surface electrons accumulate, enhancing charge-to-photon conversion.

## Conclusions

In summary, our study demonstrates the significance of surface state band transitions in two-dimensional Bi_2_Se_3_. Through time-resolved pump–probe spectroscopy and k-resolved theoretical analysis, we identified important transitions from the RSS-SS2 band that play a key role in the recombination and bleach kinetics. The contribution to the absorptance of these surface states is comparable to that involving the interior layers. The distinct structure of the bands, characterized by valleys and charge transfer points, leads to charge carrier accumulation and delayed recombination in the 2.7–3.0 eV excitation range. Our findings align with previously reported chiral photoluminescence resulting from surface state transitions and they show the potential of surface state transitions for manipulating carrier dynamics in two-dimensional materials.

## Methods and Experiments

### Chemicals

Na_2_SeO_3_ (99%, Aldrich), polyvinylpyrrolidone (*M*_w_ = 10,000, Aldrich), ethylene glycol (anhydrous, 99.8%, Aldrich), Bi(NO_3_)_3_·5H_2_O (99,999%, Aldrich), ethanol (anhydrous 99.8%, VWR chemicals), acetone (anhydrous 99.8%, VWR chemicals), and acetonitrile (anhydrous, 99.8%, Aldrich).

### Synthesis Procedure

Bi_2_Se_3_ nanoplatelets (NPLs) were synthesized as previously described in Moes et al.,^13^ except a growth time of 2 h was used. In short, 0.05 g of sodium selenite, 0.22 g polyvinylpyrrolidone (PVP) and 9.5 ml ethylene glycol were mixed in a 50 ml round-bottom flask. Here, PVP functions as a surfactant and shape and size focusing agent.^31−39^ After degassing for 15 min at room temperature, the solution was heated to 50 °C to dissolve all sodium selenite. Upon reaching 50 °C, the flask was put under N_2_ and the mixture was heated to 190 °C. At 190 °C, a Bi precursor injection solution (0.2 g Bi(NO_3_)_3_·5H_2_O in 1 ml ethylene glycol) was heated under N_2_ on a 200 °C heating plate until 30 s after its color changed to turbid white. 0.5 ml of this Bi precursor was then injected into the selenium mixture. The NPLs were left to grow at 190 °C for 2 h, after which the reaction mixture was cooled to room temperature using a water bath. The black product was transferred to a scintillation vial in a glovebox and washed by the addition of a mixture of acetonitrile and ethanol, followed by centrifugation. The resulting black precipitate was redispersed in 10 ml ethanol. The samples were stored in a glovebox under N_2_ atmosphere. Figure S23 shows STEM–EDX measurements of a NPL.

### Sample Characterization

TEM samples were prepared by drop-casting a diluted dispersion of NPLs on 200 mesh Formvar/carbon-coated Cu TEM grids. The samples were imaged using a Talos F200X (S)TEM instrument operating at 200 keV. STEM–EDX measurements were performed on an aberration-corrected thermo scientific spectra 300 (S)TEM instrument operating at 300 keV.

AFM samples were prepared by diluting the NPL stock dispersion approximately 10*x*, then sonicating this dilution for 10 min at room temperature, and subsequently drop-casting 20 μL of this dispersion onto a freshly cleaved mica surface. Measurements were carried out using a JPK Nanowizard II operating in intermittent-contact mode in an ambient atmosphere. Bruker OTESPA-R4 (300 kHz, 26 N/m) SPM tips were used to acquire images with a typical set point of 0.6 V.

UV–vis measurements were performed on a PerkinElmer Lambda 950 UV–vis–NIR spectrometer using a quartz cuvette with a path-length of 1 cm. The samples were prepared by diluting a stock dispersion of NPLs in ethanol approximately 100 times.

### Transient Absorption Measurements

Samples were measured as either a dispersion or a film. For the dispersion measurements, a dispersion of Bi_2_Se_3_ NPLs in ethanol (anhydrous, handled in GB) was diluted to obtain a stock dilution of OD 0.3 around 2.1 eV. This concentration was chosen to obtain an optimal trade-off between having a good signal and not having a too strong absorption at the pump wavelength as to ensure a uniform pumping of the sample. The sample was sealed under N_2_ in a quartz cuvette of path-length 2 mm. During the measurements, the sample was stirred continuously to avoid charging or photodegradation. Furthermore, after every few measurements, the sample was refreshed from the stock dilution. For the measurements on films, the NPL stock dispersion was diluted 10*x*, after which 50 ml was dropcast onto a 1 mm thick sapphire (Al_2_O_3_) substrate of 12.7 mm in diameter, transparent from 150 nm up to 6 mm. The thin films were prepared to obtain enough probe light for a good signal-to-noise ratio. Although the thickness of the films differs locally, the UV–vis absorbance maximum (OD) of each film was kept around 0.3. During measurements on films, the pump beam position on the sample was changed every few measurements to reduce degradation. Figure S1 shows photographs taken of representative samples.

For measurements on dispersions, the samples were excited using 110 fs pump pulses at 400 nm through second-harmonic generation in α-BBO, or at other wavelengths created from the 800 nm fundamental (Spitfire Ace, Spectra Physics) through nonlinear conversion in an optical parametric amplifier (TOPAS, Light Conversion). The probe pulses were generated in a 4 mm thick Sapphire crystal by using the 800 nm fundamental. The pulses were delayed relative to the pump using a delay stage with a maximum delay of 6 ns for TOPAS pumping and 3.3 ns for 400 nm pumping. The probe spectrum in our experiments covers the visible window from 480 to 750 nm.

For measurements on films, the samples were excited using 170 fs pump pulses at 343, 375, 400, 440, 450, 515, 525, and 1050 nm, either through second and third harmonic generation in alpha-BBO (for 515 and 343 nm, respectively) of the 1030 nm fundamental coming from a Pharos PH2 (Light Conversion) or through an OPA (Orpheus-NEO). Probe pulses were generated in a 10 mm thick YAG or sapphire crystal by using the 1030 nm fundamental. The pulses were delayed relative to the pump using a home-built spectrometer with a delay stage and a maximum delay of 6 ns. The probe spectrum in our experiments covers the spectral window from 520 nm up to 950 nm (YAG) and 440–800 nm (sapphire). For cryogenic temperature TA measurement, a cryostat CS204F-DMX-20 (Advanced Research Systems) was used to cool the sample to 9 K.

The beam area, approximated as an ellipse and obtained through a Thorlabs CCD beam profiler, is defined as *A*_beam_ = π × σ_*x*_σ_*y*_ where σ_*i*_ is the standard deviation in the *i* = *x*, *y* direction. The photon flux *J*_ph_ was calculated from the beam area by using the following formula
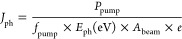
1where *P*_pump_ is the pump power, *f*_pump_ is the laser frequency divided by 2 to take into account the exposure time of the sample, *E*_ph_ is the pump energy, and *e* is the elementary charge.

### Data Processing

Data processing was performed by using a custom Python script to handle the extraction, correction, normalization, and visualization of the spectroscopic data. The data was structured with a row of time values, a column of wavelength values, and the spectroscopic data matrix in between. Extracted time values, wavelength values, and data matrices were stored in separate lists for further processing.

Wavelength values were converted from nanometers (nm) to electronvolts (eV) using the relation *E*(eV) = 1240/λ(nm). Subsequently, a background correction was performed by subtracting the mean signal for each wavelength (averaged for a number of specified time steps before *t*_0_) from the data matrix. The corrected data matrix was then normalized by dividing it by its maximum absolute value. This transformation ensured that the data was appropriately scaled for visualization in the maps. Data for traces (energy or time-resolved traces) were not normalized unless specified.

To correct for chirp, an interactive plotting feature was implemented using mpldatacursor, which enables clicking on the plot to capture points for chirp fitting. A polynomial fit (third-order) was applied to these points to model the chirp. After inspection, the polynomial fit was used to correct the time values for each data point by calculating the time correction and adjusting the original time values accordingly (i.e., by subtracting the difference between the polynomial fit value for each energy and the polynomial fit value at the first energy (*t*_0_)). This difference represents the chirp correction that needs to be applied to the original time values. A *t*_0_ correction was then performed by subtracting the value of *t*_0_ from all-time values.

To be able to plot the data, interpolation is required, as each wavelength value is associated with a different set of time values due to the chirp correction. Hence, the data was interpolated to the original time resolution using linear interpolation. After interpolation, the energy resolved traces were averaged per probe 0.1 eV to increase the S/N ratio. A Gaussian smoothing was used to reduce high-frequency noise for both the maps and the energy resolved traces while taking care not to obscure any important features. No smoothing was applied to the time-resolved traces. For plotting, a symlog scale was used for the time axis to ensure correct handling of data around time zero. A linthresh of 0.1 ps was used, with linscale = 0.25. For the fitting of the bleach decay, we use the general form of the rate equation
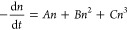
2where, in the case of free carriers, *n* is the photogenerated carrier density, *A* represents first-order recombination (Shockley–Read–Hall, or trap-mediated recombination), *B* represents second-order recombination (recombination of free carriers, where no. Electrons = no. holes), and *C* represents third-order recombination (Auger recombination).^28,40,41^ The solution for a first order process (i.e., neglecting *B* and *C*) is
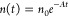
3while purely second order kinetics gives us
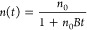
4

Nonlinear least-squares fitting was used to fit the bleach decay averaged for probe energies between 1.9 and 2.0 eV after 10 ps with the first and second order equations. For each pump energy, A and B were treated as global parameters while the carrier density *n*_0_ was varied. A weighted fitting procedure was used in which the earlier data points were given relatively more weight. The fits are shown in Figures S14 and S15 and the original curves are shown in Figure S20. The extracted fit parameters are shown in Tables 1 and 2.

**Table 1:** Extracted Fit Parameters *A* (First Order) and *B* (second Order) for First Order and Second Order Fits of the Bleach Decay after 10 ps

pump E (eV)	first-order (*A*)	second-order (*B*)
3.31	9.120 × 10^–3^	3.077 × 10^1^
3.10	8.976 × 10^–3^	1.938 × 10^1^
2.82	7.480 × 10^–3^	7.136 × 10^–1^
2.36	3.790 × 10^–3^	6.848 × 10°
1.18	6.756 × 10^–3^	1.910 × 10^1^

**Table 2:** Extracted Fit Parameter *n*_0_ for First Order and Second Order Fits of the Bleach Decay after 10 ps

pump E (eV)	fluence (Photons/cm^2^)	first-order (*n*_0_)	second-order (*n*_0_)
3.31	4 × 10^13^	1.506 × 10^–4^	1.404 × 10^–4^
3.31	8 × 10^13^	1.892 × 10^–4^	1.794 × 10^–4^
3.31	1 × 10^14^	4.652 × 10^–4^	4.932 × 10^–4^
3.10	8 × 10^13^	4.198 × 10^–4^	4.114 × 10^–4^
3.10	2 × 10^14^	6.986 × 10^–4^	7.343 × 10^–4^
2.82	2 × 10^13^	2.458 × 10^–3^	2.166 × 10^–3^
2.82	4 × 10^13^	4.976 × 10^–3^	4.590 × 10^–3^
2.82	8 × 10^13^	7.095 × 10^–3^	7.095 × 10^–3^
2.82	2 × 10^14^	9.243 × 10^–3^	9.013 × 10^–3^
2.36	4 × 10^13^	7.956 × 10^–5^	7.748 × 10^–5^
2.36	8 × 10^13^	4.557 × 10^–4^	4.530 × 10^–4^
2.36	2 × 10^14^	7.243 × 10^–4^	7.305 × 10^–4^
1.18	4 × 10^13^	1.293 × 10^–4^	1.200 × 10^–4^
1.18	8 × 10^13^	2.312 × 10^–4^	2.226 × 10^–4^
1.18	2 × 10^14^	4.788 × 10^–4^	4.913 × 10^–4^

### Computational Details

We determined the ground state properties of Bi_2_Se_3_ 6QL nanocrystals by solving the Kohn–Sham (KS) equations within the DFT formalism, using the quantum espresso code.^42,43^ All simulations have been performed employing the GGA-PBE^44^ exchange correlation functional and fully relativistic pseudopotentials from PseudoDojo.^45^ We set plane wave kinetic energy cutoff to 140 Ry using a 12 × 12 × 1 k-point grid to compute the charge density. The transition energies and oscillator strength have been computed on a 48 × 48 × 1 k-point grid. The distance between periodic replicas is 20 Å. We included spin orbit coupling and vdW corrections.^46,47^ The system has been relaxed with the Broyden (BFGS^48^) algorithm. Atomic forces and pressure were converged to within 1 × 10^–6^ Ry/Bohr, and the stress tensor components remained below 1 × 10^–4^ kbar. For neutral excitations, we utilized the Bethe–Salpeter Equation (BSE) for electron–hole interactions, exploiting the Ab-Initio Many-Body Perturbation Theory formalism implemented in the Yambo code.^49,50^ We employed the IP approximation, where optical transitions are calculated based on electronic dipoles in a single-particle framework, thus omitting many-body effects from the BSE kernel. The justification for this approach is supported by GW calculations on similar systems with varying numbers of QLs, recently published.^13^ We investigated the differences between the GW-BSE approach and IP methods for 2QL Bi_2_Se_3_ (Supporting Information, Figure S3). We observed a systematic energy shift of approximately ∼100 meVs and a renormalization of the intensities. This can be explained as follows: the GW-BSE method accounts for correlations between electrons that are missing at the single-particle DFT level. Hence, the GW approximation often increases the band gap, which improves the computed DFT gap. The BSE equation allows us to compute the excitonic energies, creating in-gap states due to the Coulomb interaction between the electron and holes. Once the GW-BSE machinery is applied, the relative shift with respect to the IP approximation depends on the two effects. The GW approximation tends to blue-shift the spectra, while the creation of exciton bound states usually results in a red-shift. In general, the net effect depends on the material, and calculations will therefore always show a discrepancy with experiment. The GW-BSE approximation also shows a difference in intensity at lower energies compared with the IP approximation. For Bi_2_Se_3_ of 2QLs, there is an increase of the band gap and an emergence of new states below the DFT electronic gap. These in-gap states are exciton states with a higher oscillator strength. Hence, the GW-BSE absorption spectra appear to be broader than the IP case.

Since the absorption bands remain well-resolved regardless of whether the IP or GW-BSE method is used, our analysis, which is based on the selected absorption bands, remains valid. Hence, we use the IP approximation, as performing GW-BSE calculations for a 6QL Bi_2_Se_3_ system would be computationally impractical.

The dielectric function is computed by summing transitions between all conduction (c) and valence (v) bands at each k-points in the BZ, according to the formula
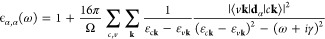
5where ε_*c*k/*v*k_ are single-particle band energies, Ω is the BZ volume, and γ is an adjustable broadening parameter.

The optical absorption spectra are computed from the imaginary part of the dielectric function ε (eq 5). We compute k-resolved absorption plots isolating the individual k-points contribution in the summation of eq 5 and integrating over a given energy range [*E*_min_, *E*_max_]. The implementation of the postprocessing routines needed to reproduce the main theoretical results of this work are available in one of the author’s (R.R.) forked repository of the Yambopy Python library.^51^

We analyzed the nature of the electronic band structure by projecting each state onto individual atoms within each QL, identifying whether electronic states are localized on the surface (surface states) or in the interior (interior states).

## Supporting Information Available

Sample photographs; AFM thickness analysis; comparison of theoretical absorption calculation methods; visualization of surface state charge density; comparison of 2D and 3D band structures; additional k-resolved absorptance maps; schematic diagrams illustrating carrier dynamics; analysis of bandgap renormalization effects; temperature-dependent transient absorption data; comparison of transient absorption in film versus dispersion; kinetic traces for various pump energies, probe energies, and fluences; kinetic fitting results; comparison of transient absorption for fresh versus photodamaged samples; and STEM–EDX analysis (PDF)
