# Supplementary material for: Role of Surface Bands in the Photogeneration, Cooling, and Recombination of Charge Carriers in Two-Dimensional Bi2Se3
Source: ACS Nano. 2025 May 5;19(18):17261–72. doi: 10.1021/acsnano.4c14134 (PMC12080342; doi:10.1021/acsnano.4c14134)
Supplement: Supplementary file 1 — nn4c14134_si_001.pdf [file nn4c14134_si_001.pdf]

Supporting information for:

Role of surface bands in the photogeneration, cooling, and recombination of charge carriers in two-dimensional Bi_2Se_3

Jara F. Vliem,[†] Servet Ataberk Cayan,[‡,¶] Riccardo Reho,[†] Andrés R. Botello-Méndez,[†] Pieter Geiregat,[‡,¶] Zeila Zanolli,[†] Daniel Vanmaekelbergh[*,†]

[†]*Debye Institute for Nanomaterials Science, Utrecht University, Princetonplein 1, 3584 CC Utrecht, The Netherlands*

[‡]*Physics and Chemistry of Nanostructures, Ghent University, 9000 Ghent, Belgium*

[¶]*NOLIMITS Center For Non-Linear Microscopy and Spectroscopy, Ghent University, 9000 Ghent, Belgium.*

E-mail: d.vanmaekelbergh@uu.nl

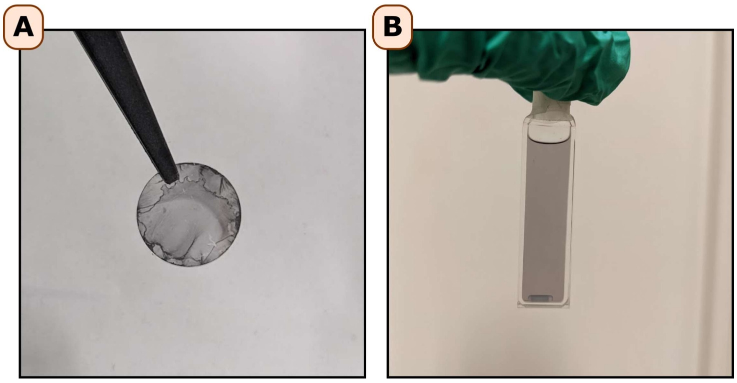

Fig. S1: **Representative samples used for pump-probe measurements.**
Photo of A) a drop-cast film on a quartz substrate and B) a dispersion of Bi_2Se_3 NPLs in ethanol.

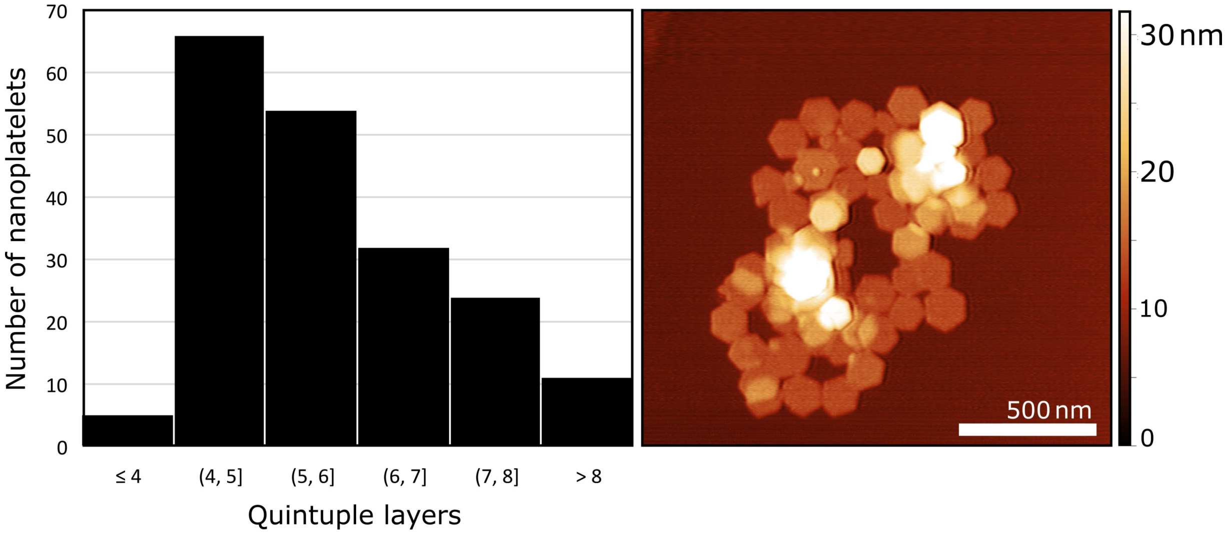

Fig. S2: **AFM on Bi_2Se_3 nanoplatelets.**
Thickness distribution in quintuple layers, assuming an integer number of quintuple layers of 0.96 nm thick.[1] The average thickness is 6.2±1.5 QLs. The data was obtained by measuring 192 Bi_2Se_3 NPLs of 4 different batches (used for TA experiments) with atomic force microscopy (AFM). A representative AFM image is shown on the right. As the surfactant coverage of the NPLs is unknown, their effect on the measured thickness was excluded, which may result in an overestimated thickness.

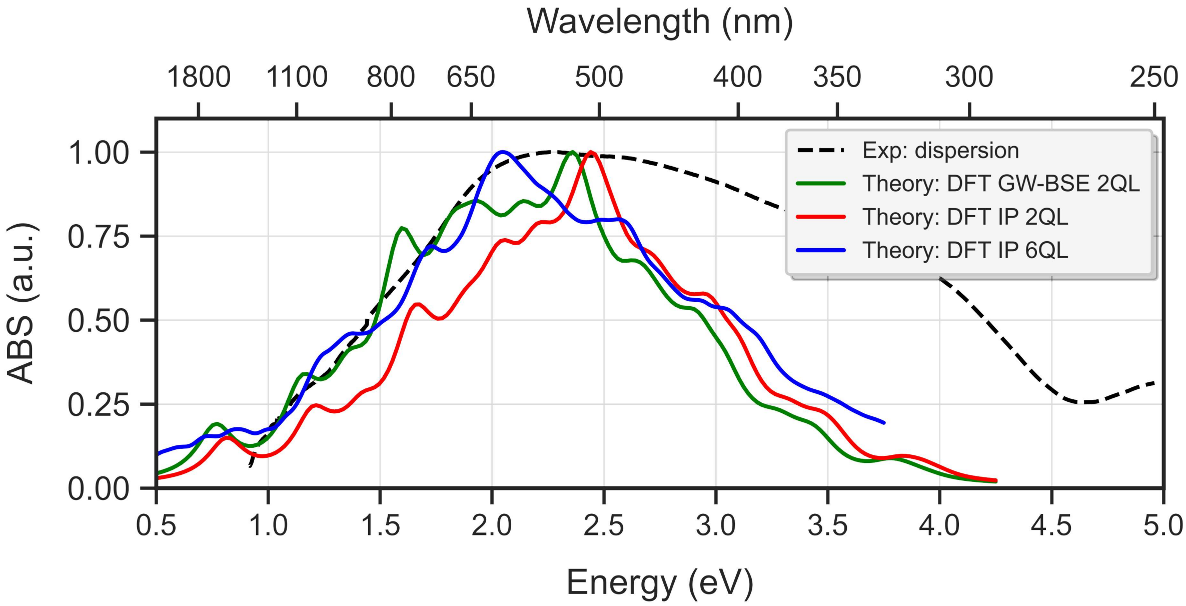

Fig. S3: **IP versus GW-BSE approach, compared to experimental absorbance.** Absorptance spectra for an infinite Bi_2Se_3 slab of 2 QLs, computed with the GW-BSE approach (green) and the Independent Particle (IP) approximation (red). The blue spectrum was computed with the IP approximation for a laterally infinite Bi_2Se_3 slab of 6 QLs. All theoretical spectra were blueshifted 0.25 eV to match the experimental absorbance (black dashed line).

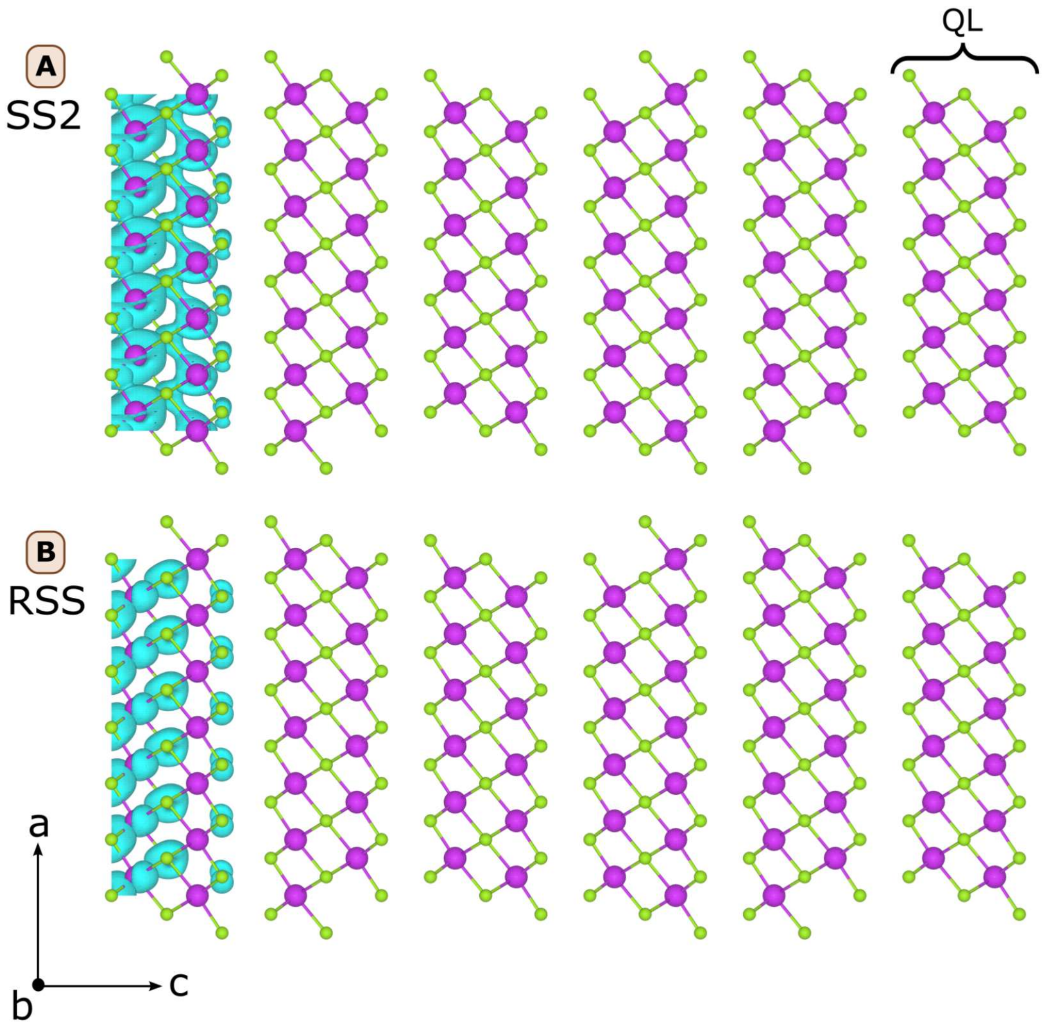

Fig. S4: **Visualization of the contribution of a surface state in the atomic structure of 6 QL Bi_2Se_3 to the charge density**. Contribution of the SS2 (A) and RSS (B) states to the charge density. This contribution is computed for a **k**–point along $\Gamma - K$ with high surface contribution. The figure shows only one of the two energy-degenerate surface states. The paired surface state is located on the rightmost layer. a, b and c are the unit cell vectors.

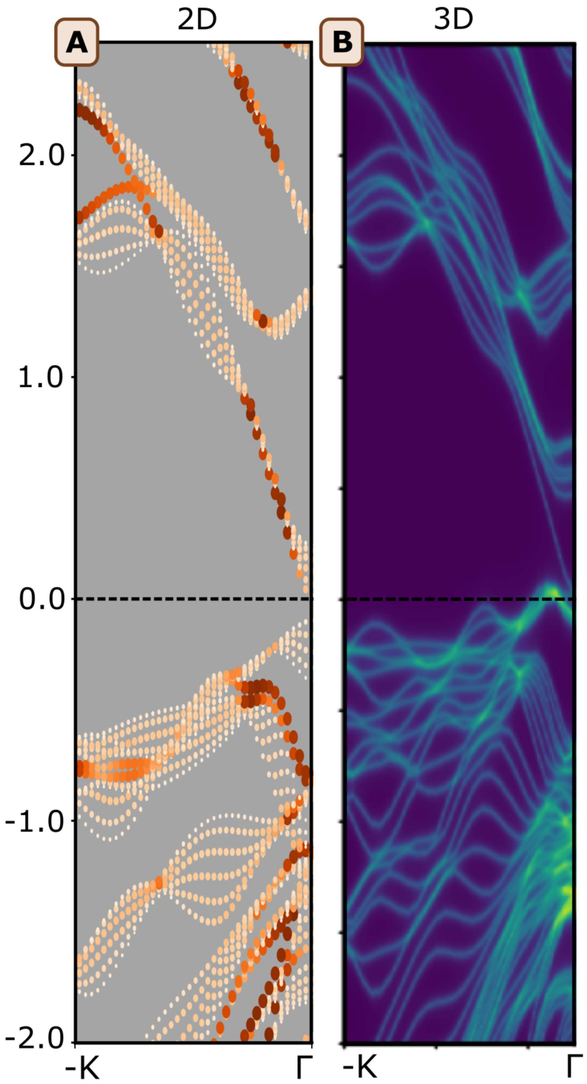

Fig. S5: **Comparison of the band structure of 2D and 3D Bi_2Se_3.**
Band structure of an infinite 6 QL Bi_2Se_3 slab calculated with DFT as described in the Computational details (A), and the 3D band structure reported by Kung *et al.* (B).[2] The band structures are very similar, except for the gapping of surface state bands around the Fermi energy and at 1.7 eV above the Fermi energy in the 2D case. The similarity between the band structures of 2D and 3D Bi_2Se_3 suggests nearly identical optical transitions in the high-energy range. This similarity is attributed to the layered structure of Bi_2Se_3, where the band structure is primarily determined by atomic interactions within a QL, with limited contributions from inter-QL interactions due to the separation by a van der Waals gap.[3]

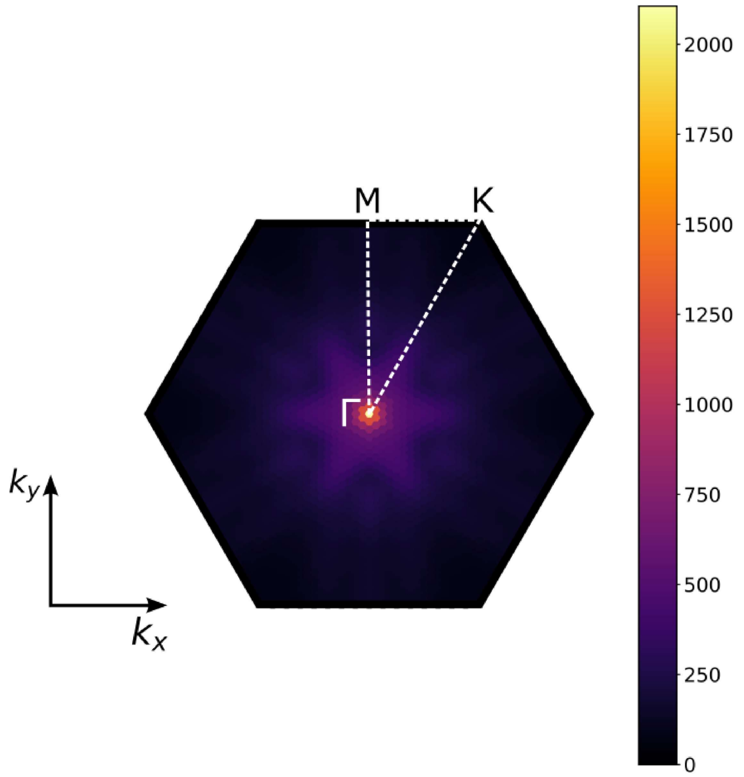

Fig. S6: **k-resolved absorptance map for E=0.0-3.0 eV.**
k-resolved map showing the integrated absorptance (color scale, arbitrary units) between $E = 0.0{-}3.0\,\mathrm{eV}$, projected onto the (E=0, k_x, k_y) plane (2D Brillouin zone).

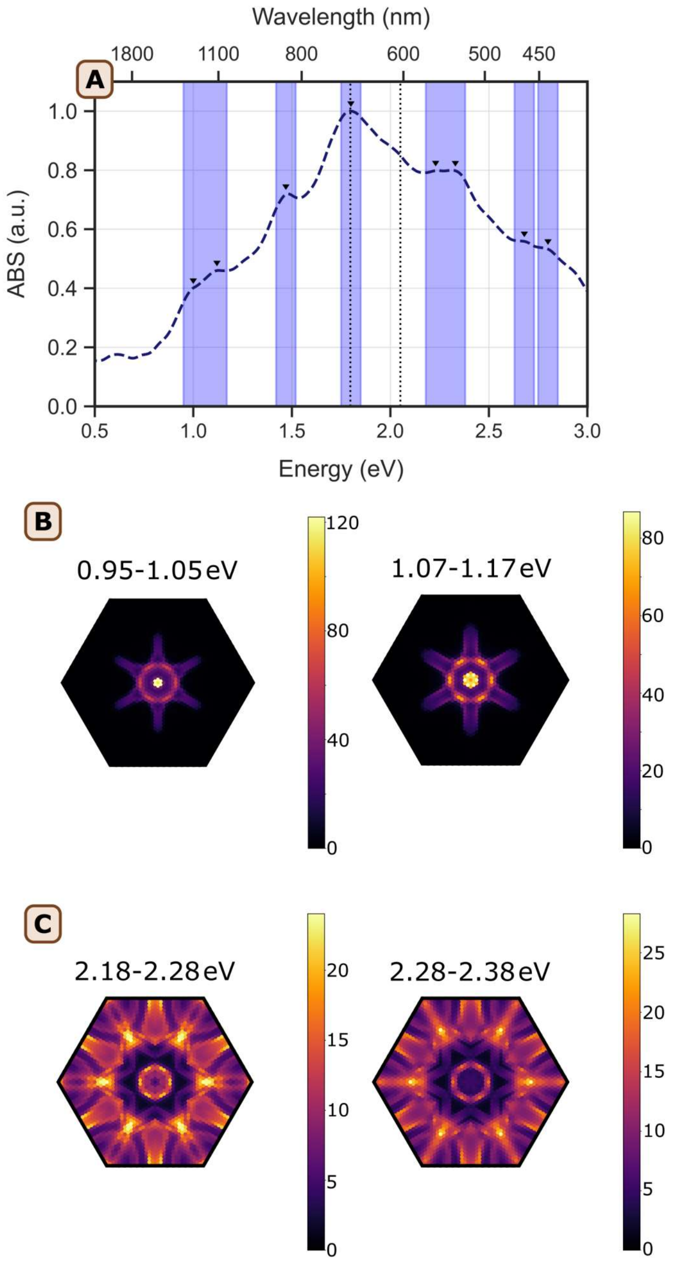

Fig. S7: **Integration ranges selected for k-resolved absorptance maps.**
(A) computed absorptance for an infinite Bi_2Se_3 slab of 6 QLs. The black markers highlight specific transitions at 1, 1.12, 1.47, 1.8, 2.23, 2.33, 2.68, and 2.80 eV. The shaded blue regions indicate the energy ranges over which the k-resolved absorptance maps presented in Figure 4 were integrated. Some consecutive transitions were grouped together due to the qualitative similarity of their k-resolved absorptance maps. These are the transitions centered around 1 and 1.12 eV, and 2.23 and 2.33 eV. Their k-resolved absorptance maps are shown in B) and C) respectively. The transitions at 2.68 and 2.80 eV were analyzed separately due to significant differences in the k-resolved absorptance near the Γ point.

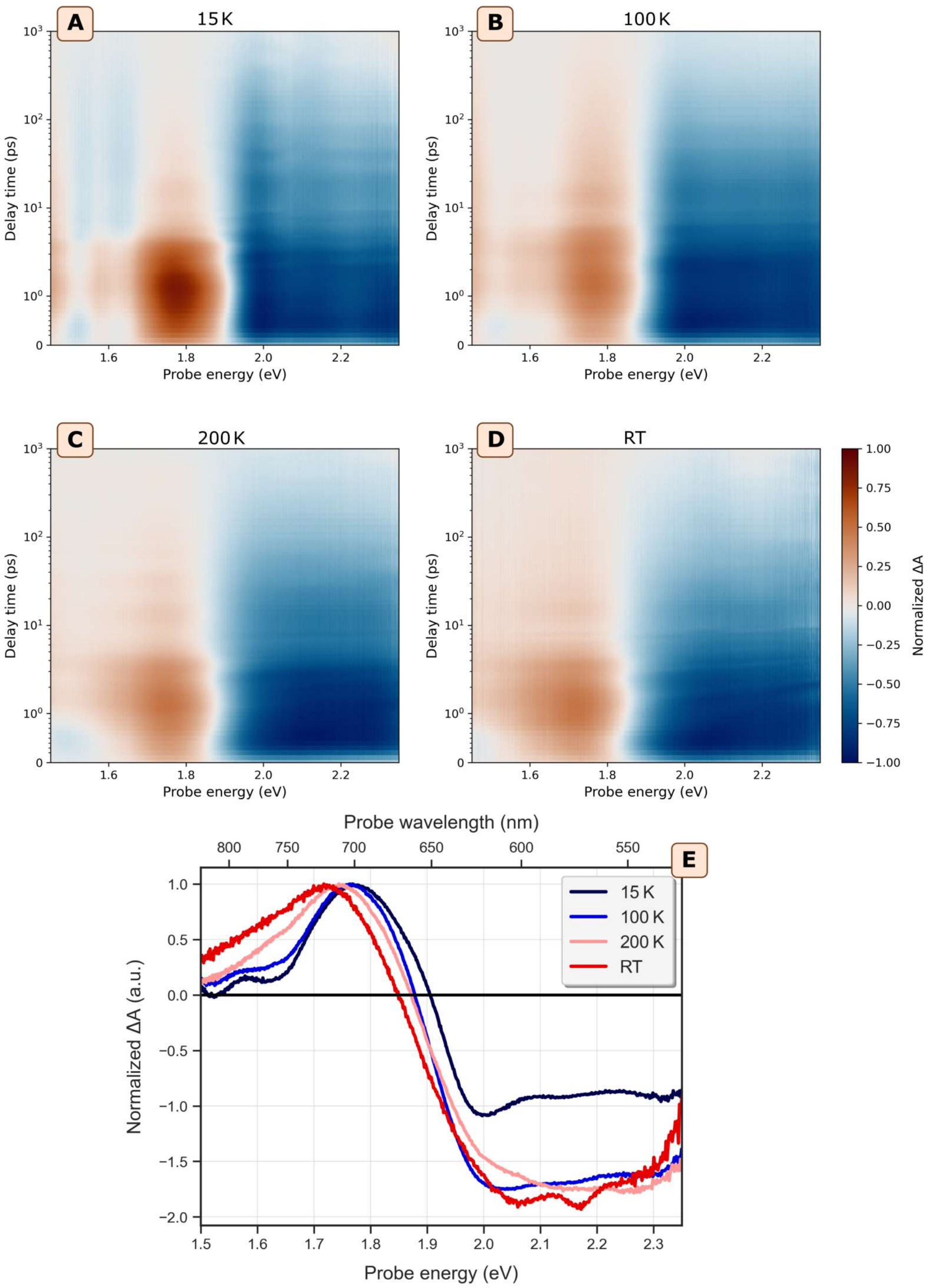

Fig. S8: **Pump(2.82 eV)-probe experiments at RT, 200, 100, and 15 K.**
A-D) Transient absorption maps showing the normalized ΔA of a dropcast Bi_2Se_3 NPL film at 15 K - RT, after photo-excitation at 2.82 eV (440 nm). E) The transient bleach spectrum at 1.5 ps for RT, 200, 100, and 15 K. A pump power of approximately 450 μW was used for each measurement, which translates to a fluence of 4.5×10^{13} photons/cm^2.

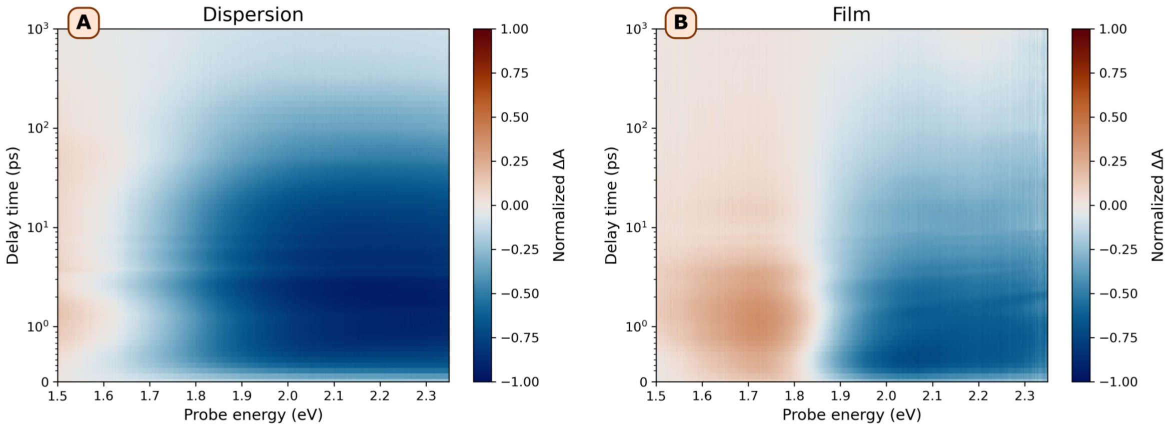

Fig. S9: **Pump(2.82 eV)-probe experiment on a film and dispersion of Bi_2Se_3 nanoplatelets.**
Transient absorption maps for dispersion (A) and film (B) showing the normalized ΔA. A pump wavelength of 2.82 eV (440 nm) was used, with a fluence of 5×10^{13} photons/cm^2. The measurements were performed at room temperature. The transient absorption data for solutions and films exhibit similar features, including a PA signal below 1.7-1.8 eV and a pronounced bleach at higher energies. The main difference lies in the transition point between PA and bleach. We attribute the different transition point to experimental factors: we note that a higher pump fluence results in a stronger bleach signal and a broader bleach region (see also Figure 7 and S21). Similarly, a difference in nanoplatelet concentration between samples could have a comparable effect, as it changes the number of charge carriers per nanoplatelet. Because the trends observed in bleach behavior over time are consistent between solution and film (see Figure S18, we conclude that the differences between film and dispersion measurements are mainly due to experimental conditions rather than sample properties.

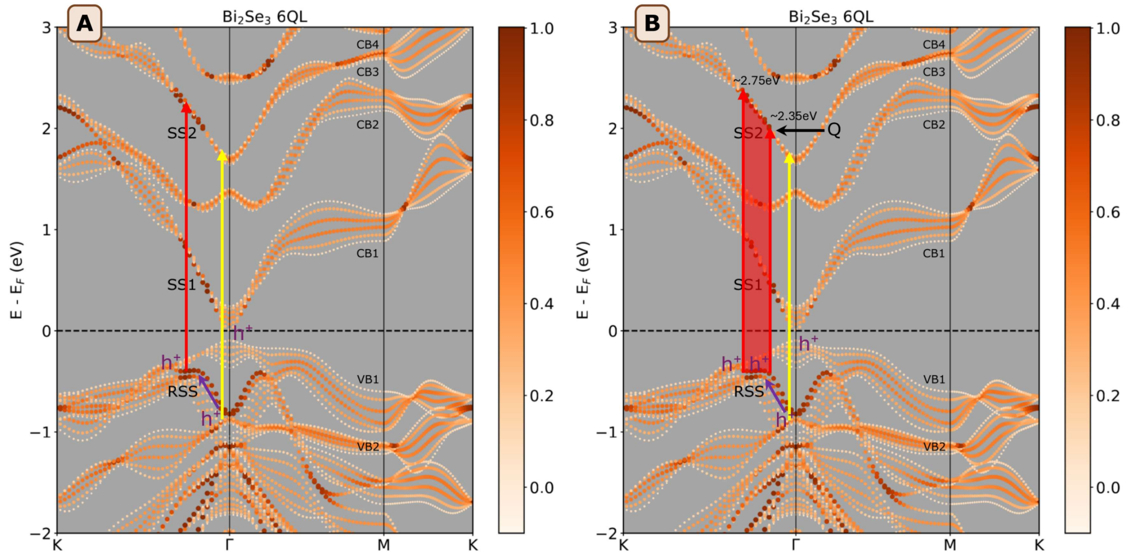

Fig. S10: **Band structure schemes illustrating transitions and movement of charge carriers.**
A) Band diagram illustrating the transitions and movement of charge carriers upon experimental excitation at 2.82 eV. The colour of the arrows corresponds to the groups the transitions belong to (red = RSS transitions, yellow = transitions around Γ). h^+ indicates positions of holes, and purple arrows indicate the movement of holes along the bands. B) Band diagram illustrating the transitions and movement of charge carriers after experimental excitation between 2.7-3.0 eV. The shaded red region is used to indicate all transitions within that range are RSS→SS2 transitions. Q is the charge transfer point at which electrons would need to transfer to the interior layers in order to relax further to Γ.

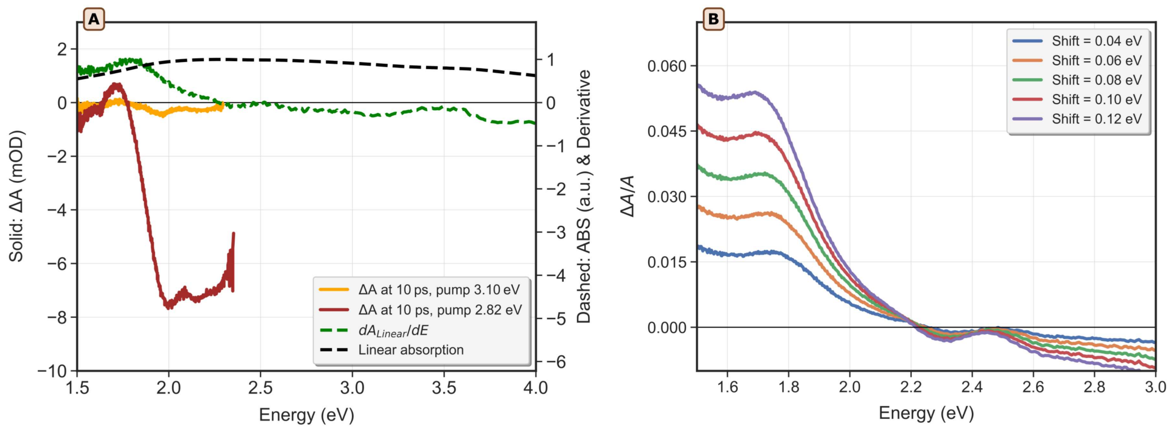

Fig. S11: **Influence of bandgap renormalization on TA spectra.**
A) Normalized linear absorption (dashed black), the derivative of this curve with respect to energy (dashed green), and two transient bleach spectra at 10 ps pump-probe delay time. The TA data were measured at 9-15 K, after photo-excitation at 3.10 and 2.82 eV with a fluence of 8×10^{13} photons/cm^2. The shift between the maximum of the green and red curves amounts to 0.078 eV. B) $\Delta A/A$ curves providing an estimate of the contribution from bandgap renormalization to the photo-induced absorption signal. The curves were generated by red-shifting the linear absorption spectrum with values between 0.04-0.12 eV, i.e. the same order of magnitude as the shift found in (A), and then dividing the difference between the shifted and original absorption spectra by the original absorption spectrum. We observe that the PA signal roughly resembles the derivative of the linear absorption spectrum. Additionally, redshift-blueshift dynamics in the PA at longer delay times could be consistent with BGR effects.[4,5] However, we also observe that the pump fluence is important here; upon comparing Figure 5C and 6A, we find that the PA signal can be entirely suppressed to a bleach signal at high excitation fluences, which is inconsistent with an expected stronger BGR contribution at higher charge carrier densities. Concerning the experimental bleach signal, we should remark that this bleach occurs in a photon energy region between 1.9-2.4 eV, while BGR should result in a positive signal below 2.2 eV and a small negative signal above 2.4 eV. The effect of BGR on the bleach can thus be neglected.

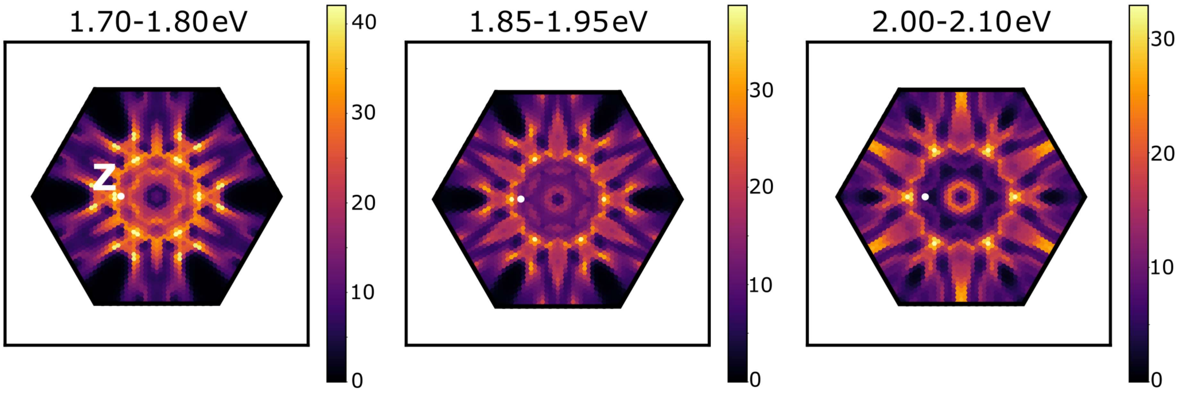

Fig. S12: **k-resolved absorptance maps integrated for strong experimental bleach bands.**
Maps of the k-resolved absorptance for energy regions centered around 2.0, 2.15, and 2.30 eV (i.e. the theoretical energies that correspond to the strongest bleach observed experimentally at pump 2.82 eV). The integration range is given in the title. The maps show where in the Brillouin zone the strongest transitions occur as indicated by the colour scale (integrated absorptance (a.u.)). As a visual aid, a Z point (i.e. the end of the RSS bands on the Γ-K line) is shown as a white dot in all plots.

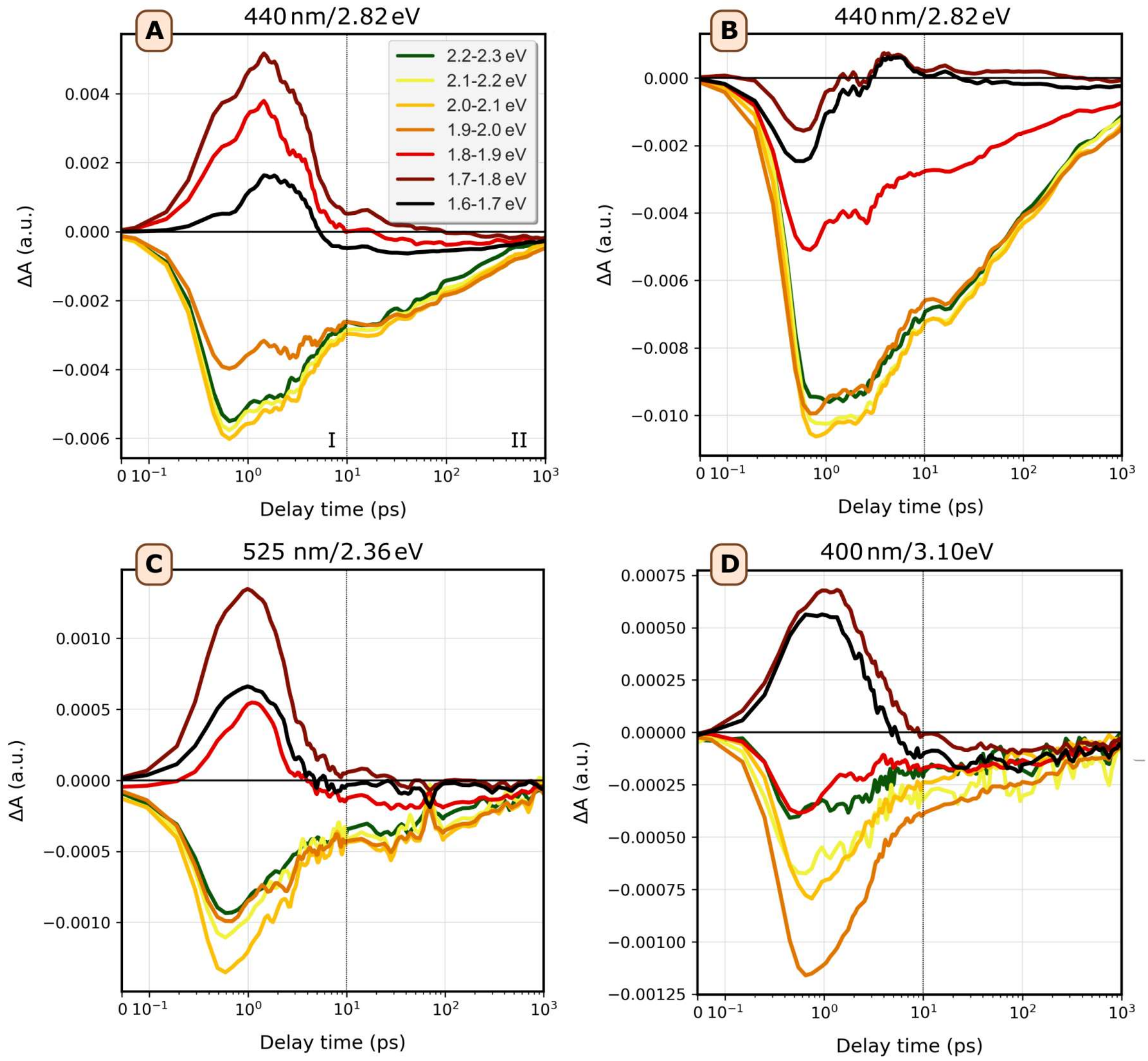

Fig. S13: **Decay kinetics of a film of Bi_2Se_3 nanoplatelets at various pump energies.** Traces of ΔA as a function of pump-probe delay time for a drop-cast Bi_2Se_3 NPL film measured at 15 K, after photo-excitation at 3.10, 2.82, and 2.36 eV. The data are averaged over each 0.1 eV between probe energies of 1.6 and 2.3 eV to reduce noise. The dotted line around 10 ps divides the figure into regime I and II, which show different kinetics. In A), a pump power of 426 μW was used, which translates to a fluence of 4.2×10^{13} photons/cm^2. For the measurements in B, C, and D, a fluence of 8×10^{13} photons/cm^2 was used.

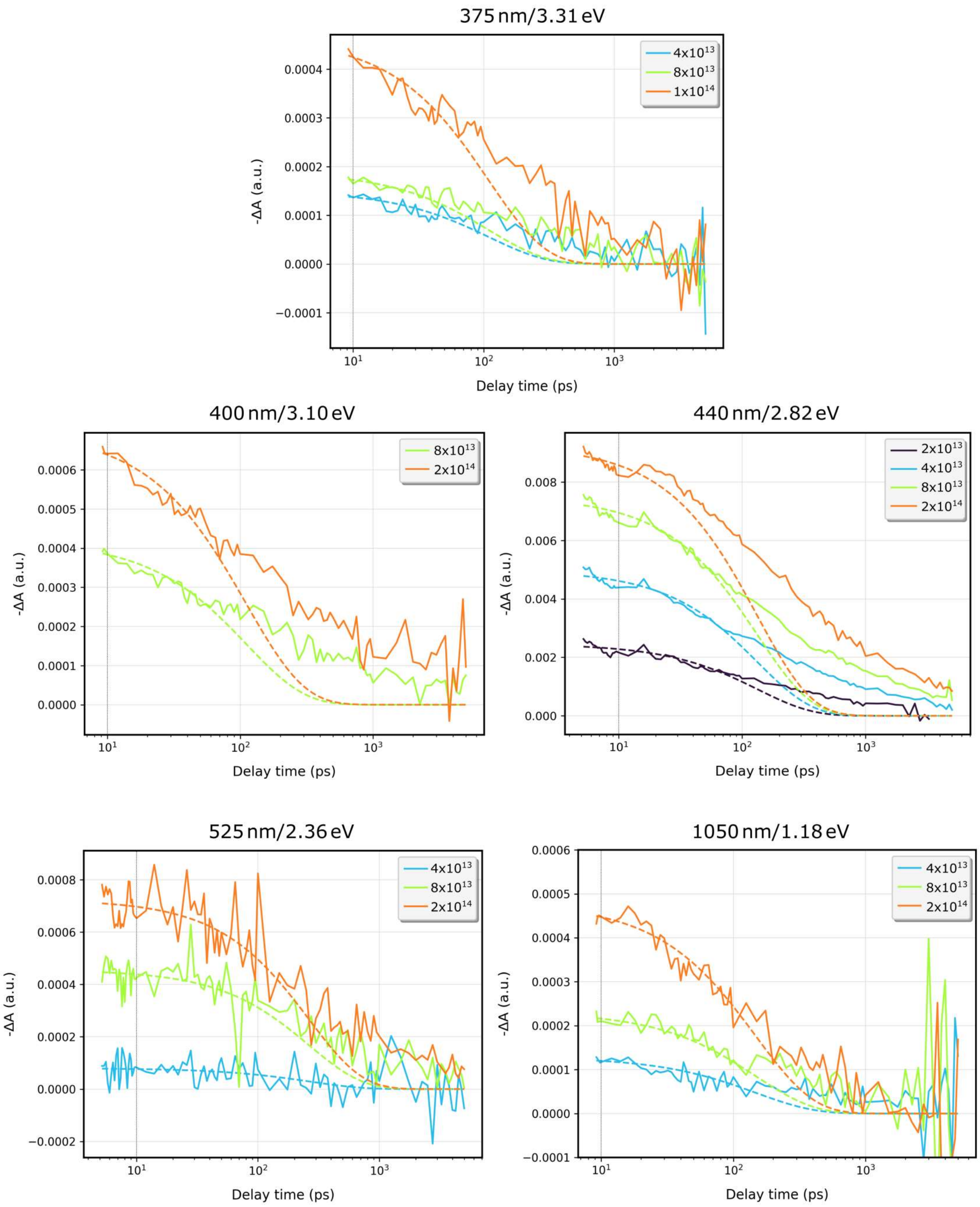

Fig. S14: **First order fits of bleach decay averaged for probe energies between 1.9-2.0 eV.**
First order fits for the bleach decay after 10 ps. The fluence in photons/cm^2 is indicated in the legend and the pump energy is indicated in the title of the plots.

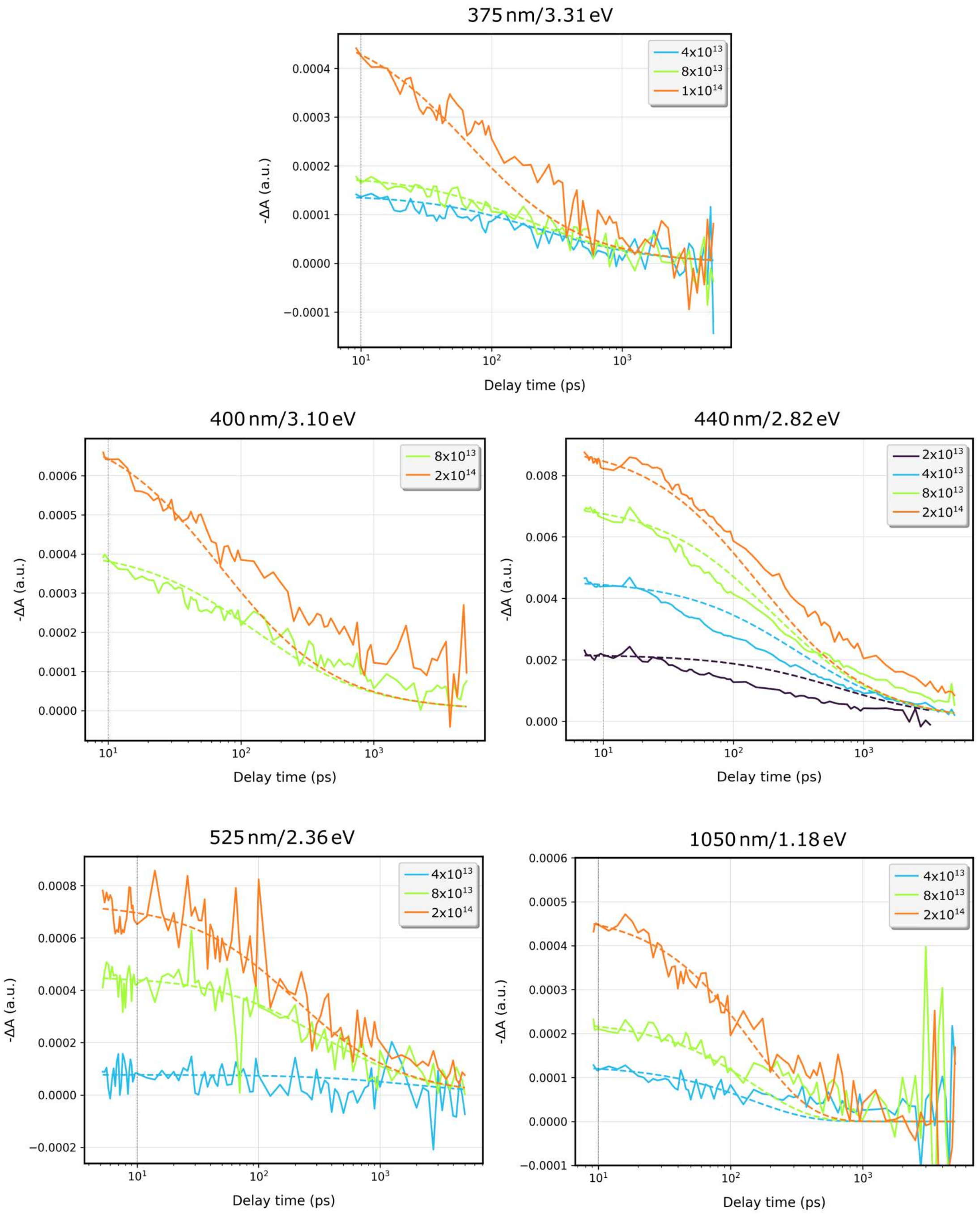

Fig. S15: **Second order fits of bleach decay averaged for probe energies between 1.9-2.0 eV.**
Second order fits for the bleach decay after 10 ps. The fluence in photons/cm^2 is indicated in the legend and the pump energy is indicated in the title of the plots.

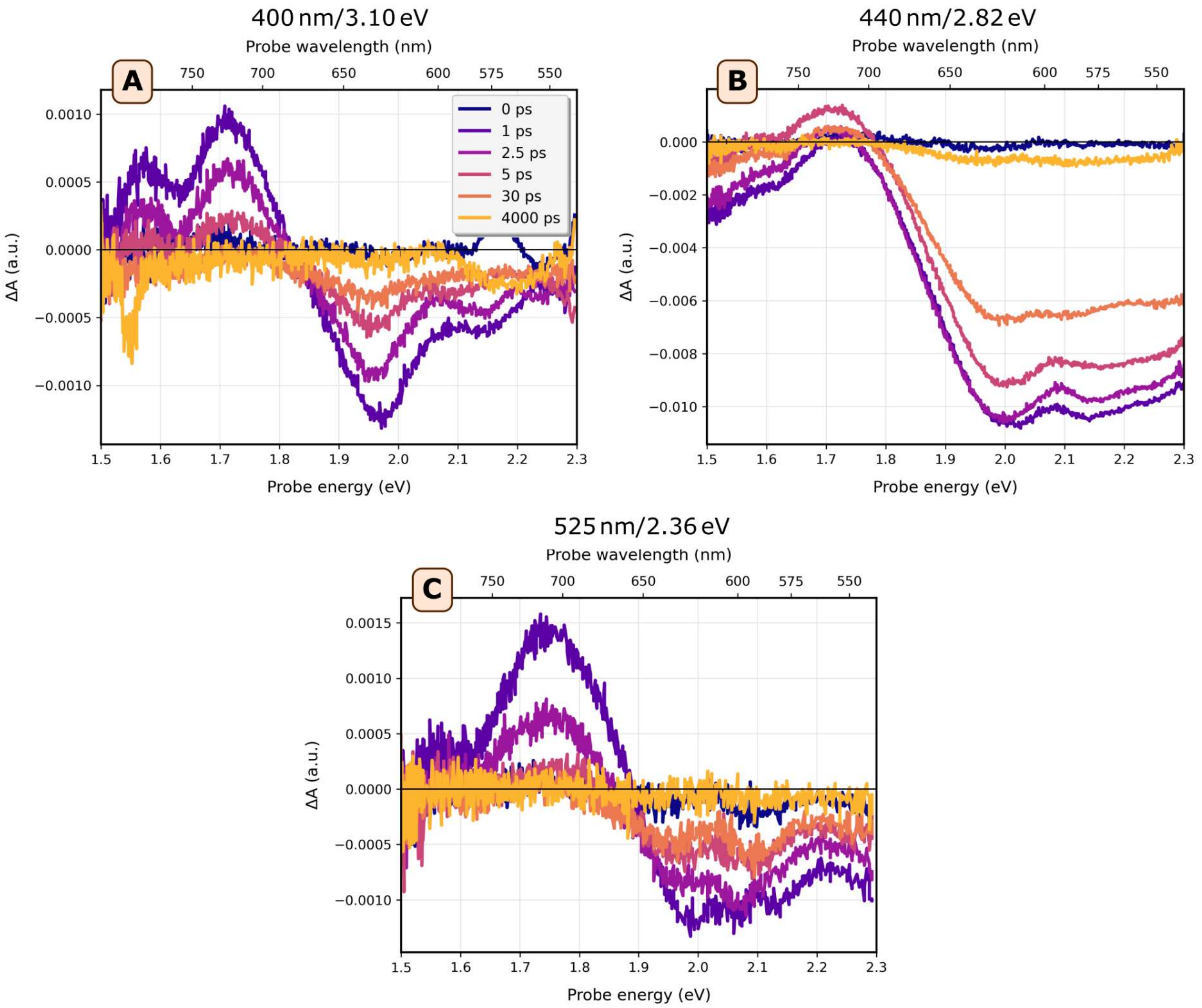

Fig. S16: **Transient bleach for films of Bi_2Se_3 nanoplatelets at various pump energies.**
The transient bleach spectrum at various pump-probe delay times measured at 9-15 K, after photo-excitation at 3.10, 2.82, and 2.36 eV. The fluence was set to 8×10^{13} photons/cm^2 for all measurements.

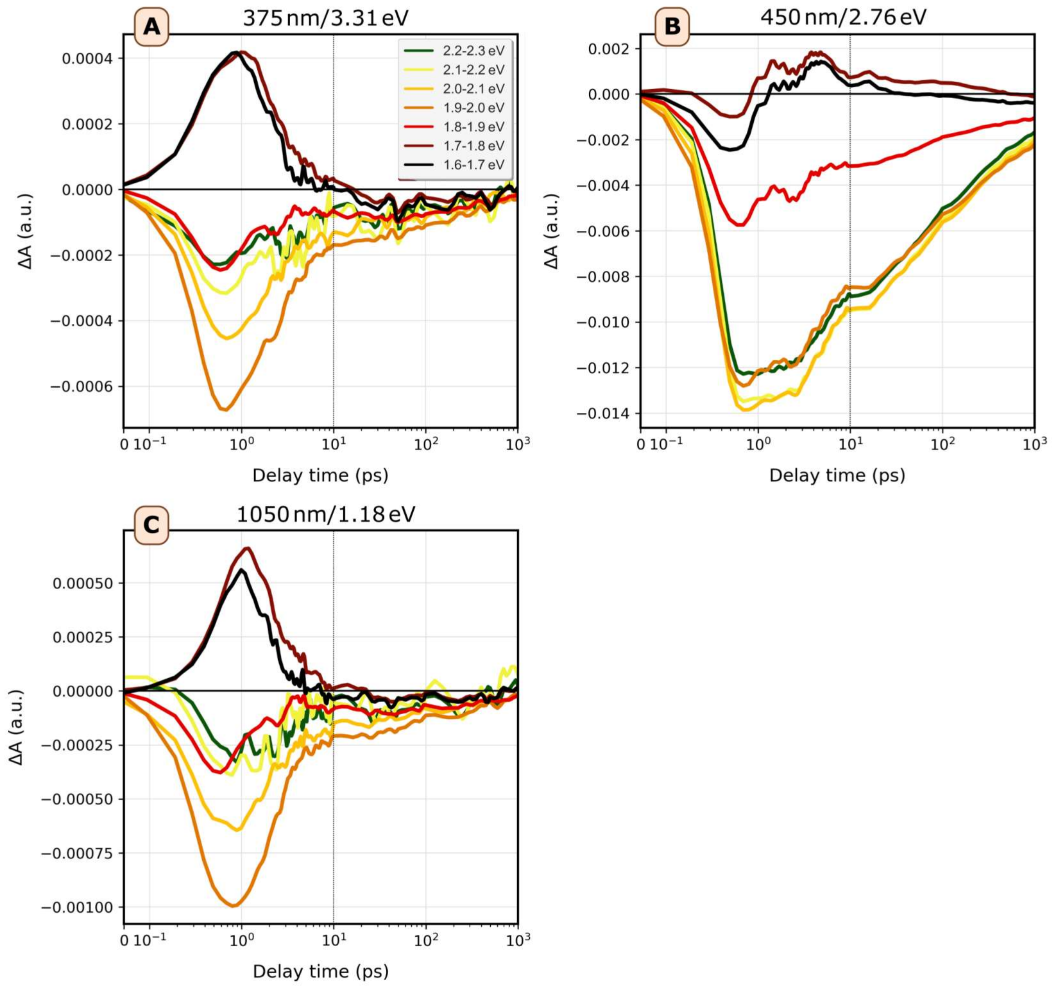

Fig. S17: **Pump-probe experiments on films of Bi_2Se_3 nanoplatelets for various pump energies at 15 K.**
ΔA as function of delay time, averaged for each 0.1 eV between probe energy 1.6-2.3 eV to reduce noise. The probe energy over which the traces are averaged is indicated with colors in the legend of (A). Pump energies of 3.31, 2.76, and 1.18 eV were used. The fluence was set to 8×10^{13} photons/cm^2 for all measurements.

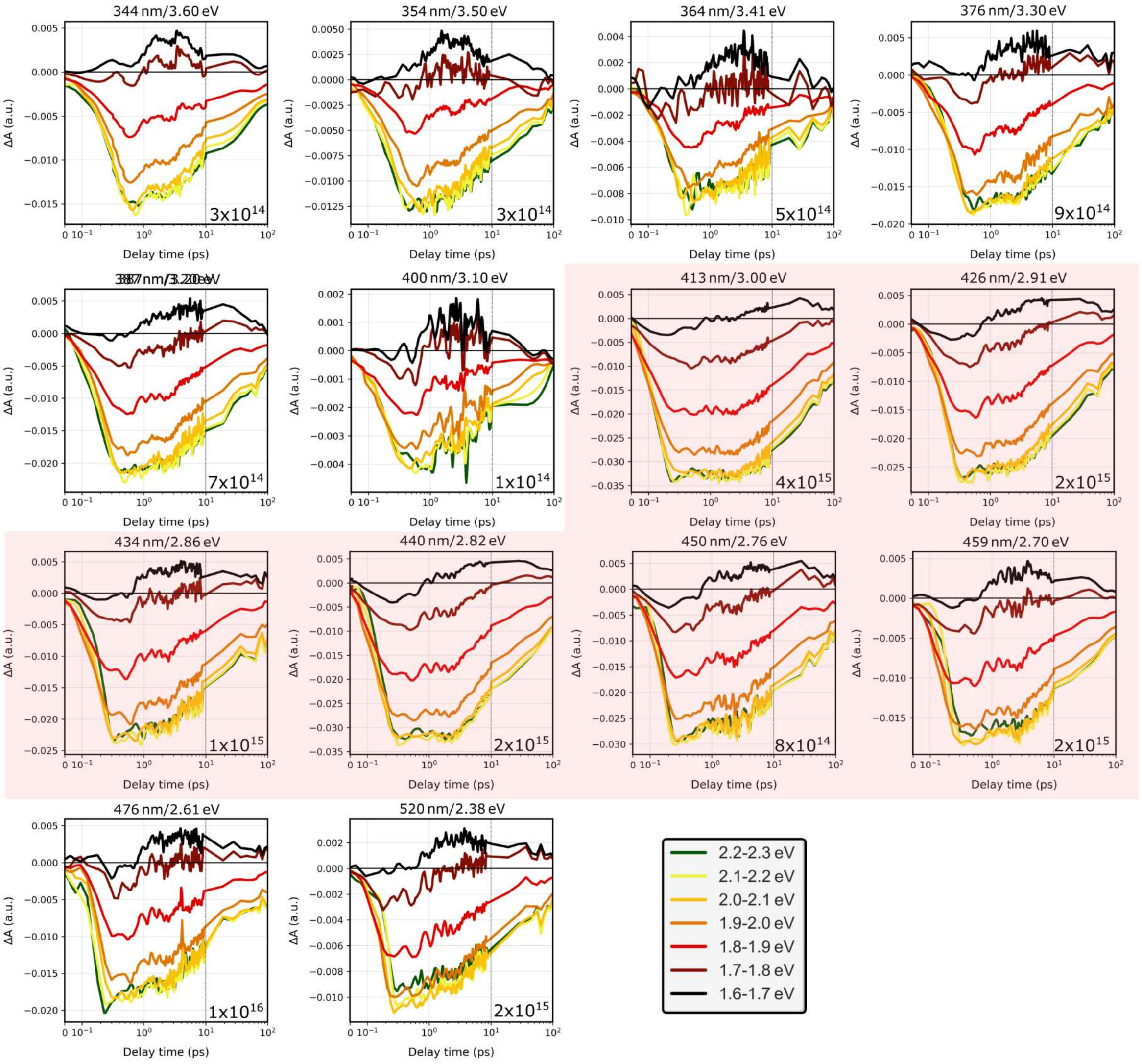

Fig. S18: **Energy dependence of pump-probe measurements for dispersions of Bi_2Se_3 nanoplatelets.**

ΔA as function of delay time, averaged for each 0.1 eV between probe energies of 1.6 and 2.3 eV to reduce noise. The excitation energy is indicated in the title of each graph, and the probe energy over which the traces are averaged is indicated by the colors in the legend. Note that the fluence, which affects the recombination kinetics, differs slightly for each measurement as indicated in right-bottom of each graph. The red box includes the graphs within the range of pump energies for which delayed recombination in regime I (<10 ps) is most prominently observed.

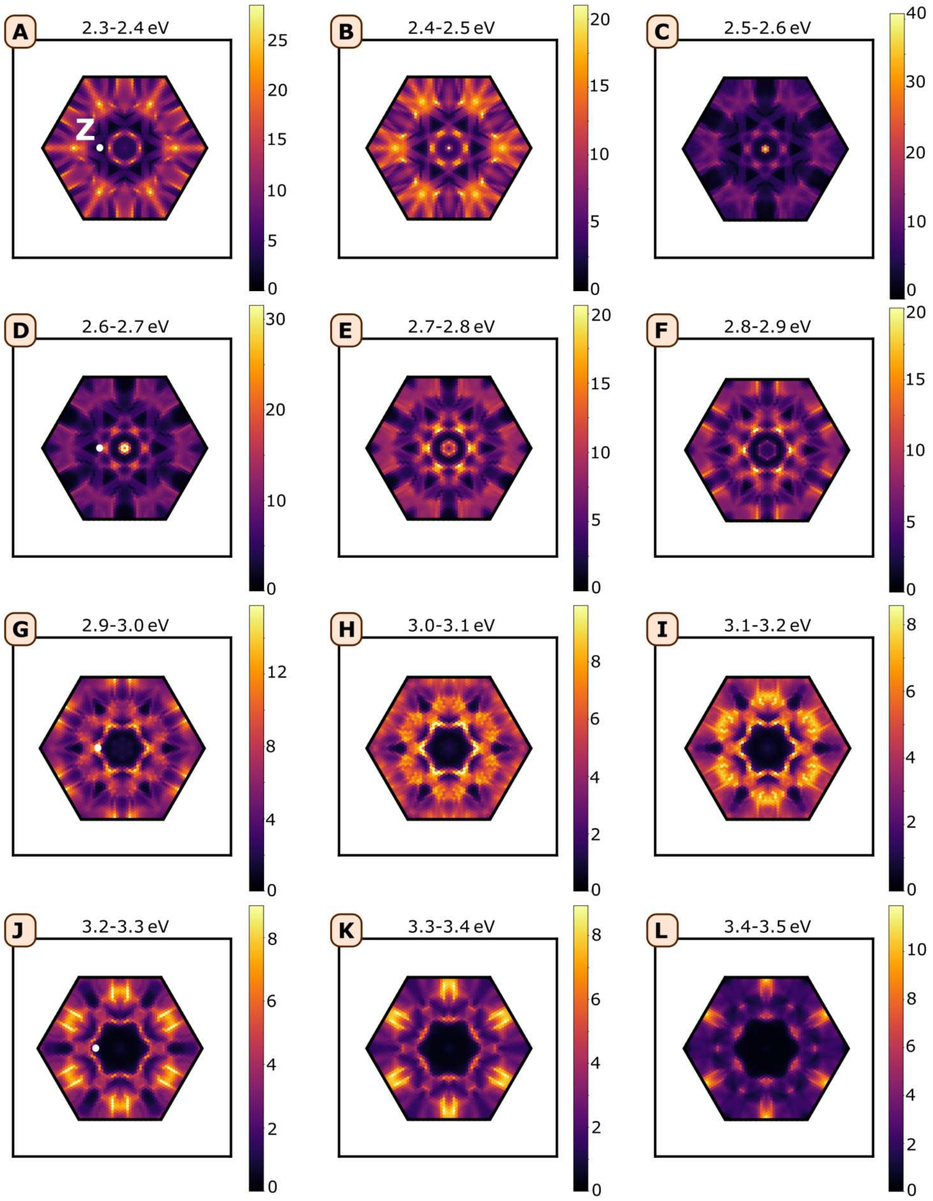

Fig. S19: **k-resolved absorptance maps integrated over energy regions between 2.3-3.5 eV.**
k-resolved absorptance maps between E=2.3-3.5 eV integrated in 0.1 eV intervals, projected onto the 2D Brillouin zone. The plots show where in the Brillouin zone the strongest transitions occur as indicated by the color scale (integrated absorptance (a.u.)). As a visual aid, a Z point (i.e. the end of the RSS bands on the Γ-K line) is shown in white for the k-resolved plots in the first column.

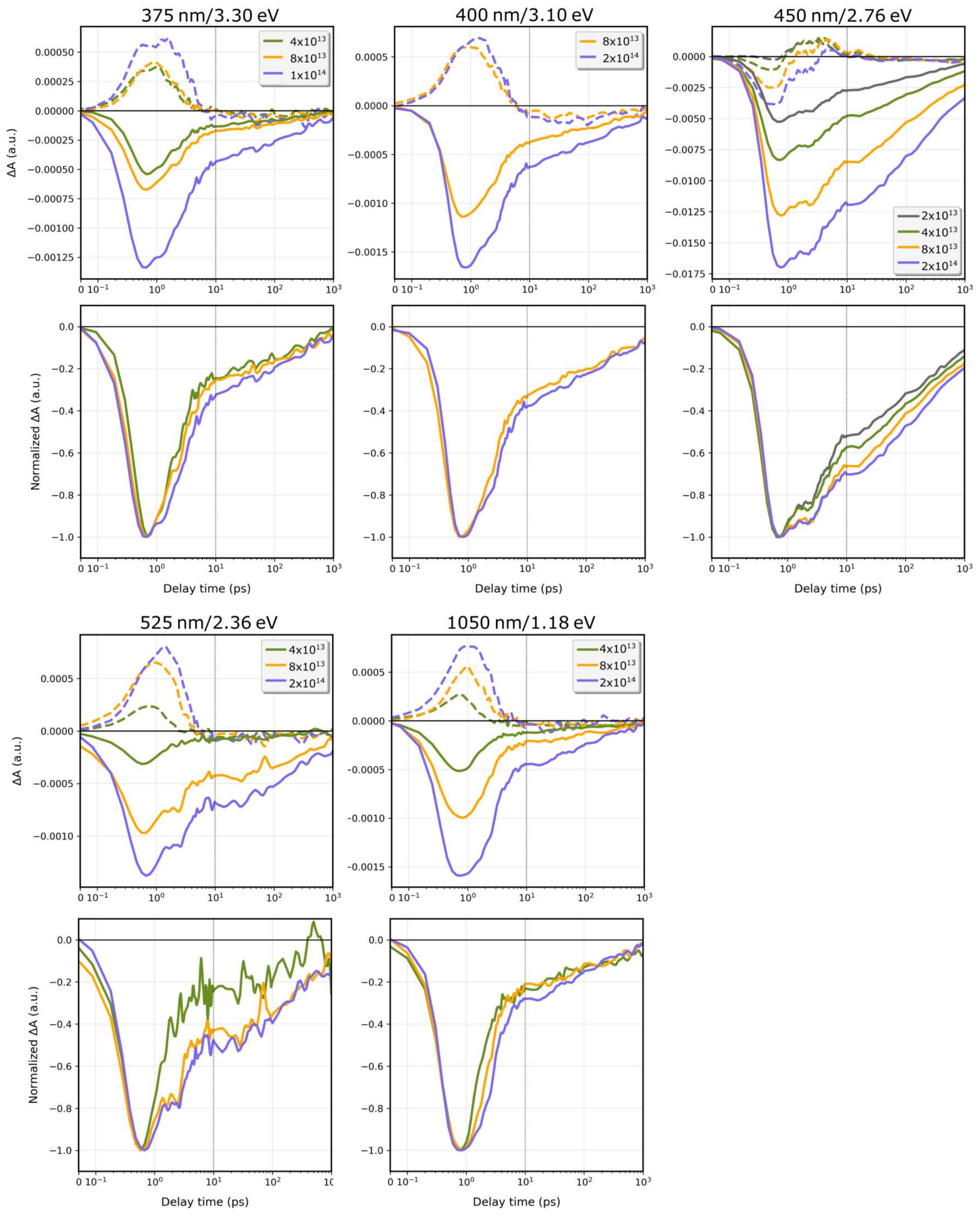

Fig. S20: **Fluence dependence of pump-probe experiments on films of Bi_2Se_3 nanoplatelets.**

For pump energies of 3.31, 3.10, 2.76, 2.36. and 1.18 eV, ΔA as function of delay time averaged between probe energies of 1.9 and 2.0 eV at various fluences (photons/cm^2). The bottom graphs show the normalized bleach traces for easier comparison. The measurements were performed at 10 K.

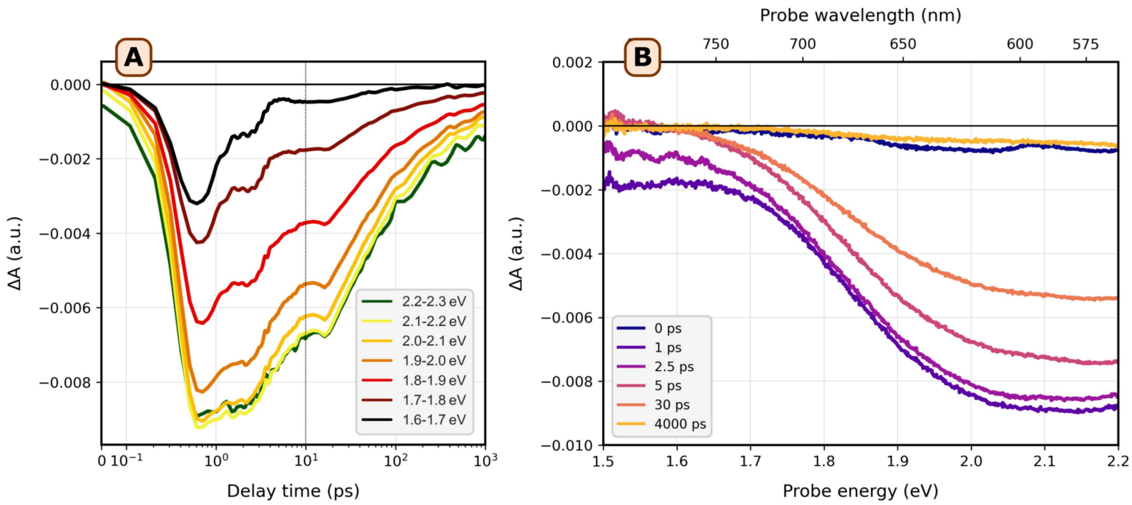

Fig. S21: **High fluence pump (2.36 eV)-probe experiments on films of Bi_2Se_3 nanoplatelets at RT.**
A) ΔA as function of delay time, averaged for each 0.1 eV between probe energies of 1.6 and 2.3 eV. B) The transient bleach spectrum at various pump-probe delay times. A fluence of 1×10^{15} photons/cm^2 was used for the measurements.

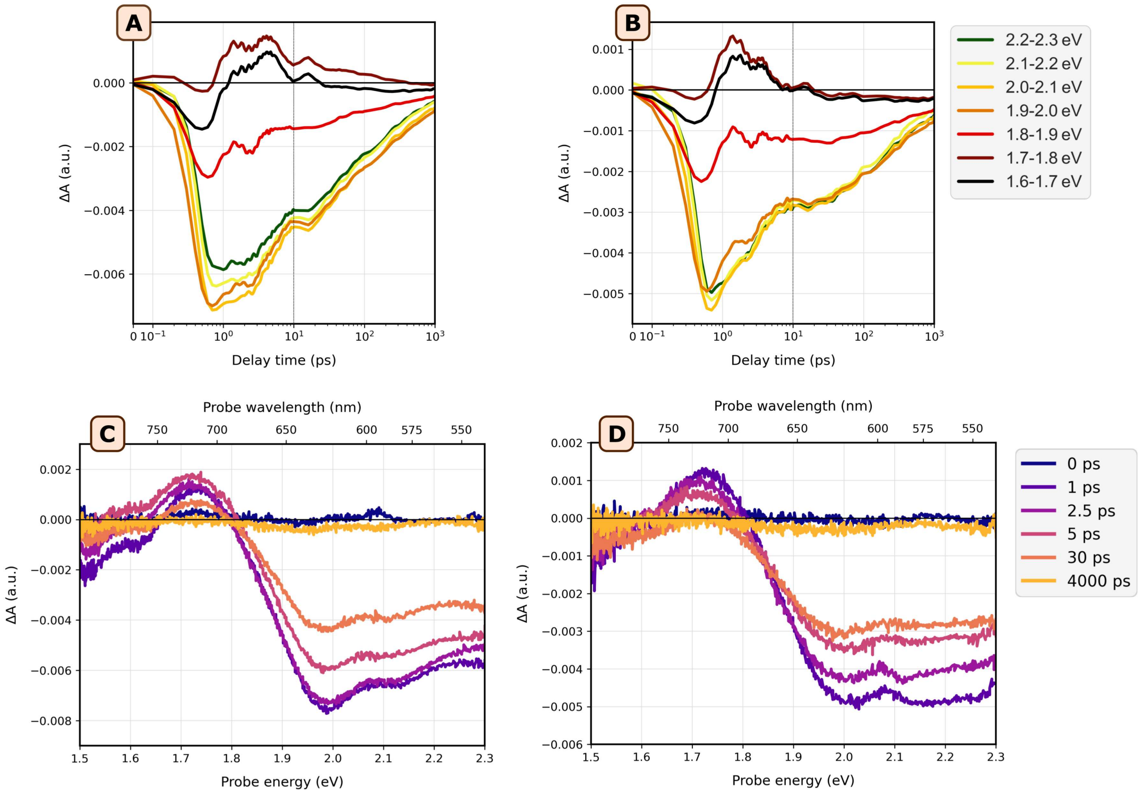

Fig. S22: **Comparison of as-prepared and photo-damaged samples in pump-probe spectroscopy.**

A,B) ΔA as function of delay time, averaged for each 0.1 eV between probe energies of 1.6 and 2.3 eV. A) shows results of the second measurement in a series, while B) shows results of the same experiment after 6 additional measurements with various pump energies and fluences had been completed. C,D) The transient bleach spectra at various pump-probe delay times, corresponding to the measurements in A,B respectively. Measurements were performed on a drop-cast Bi_2Se_3 NPL film at 9 K, after photo-excitation at 2.82 eV. A fluence of 3.8×10^{13} photons/cm^2 was used for both measurements.

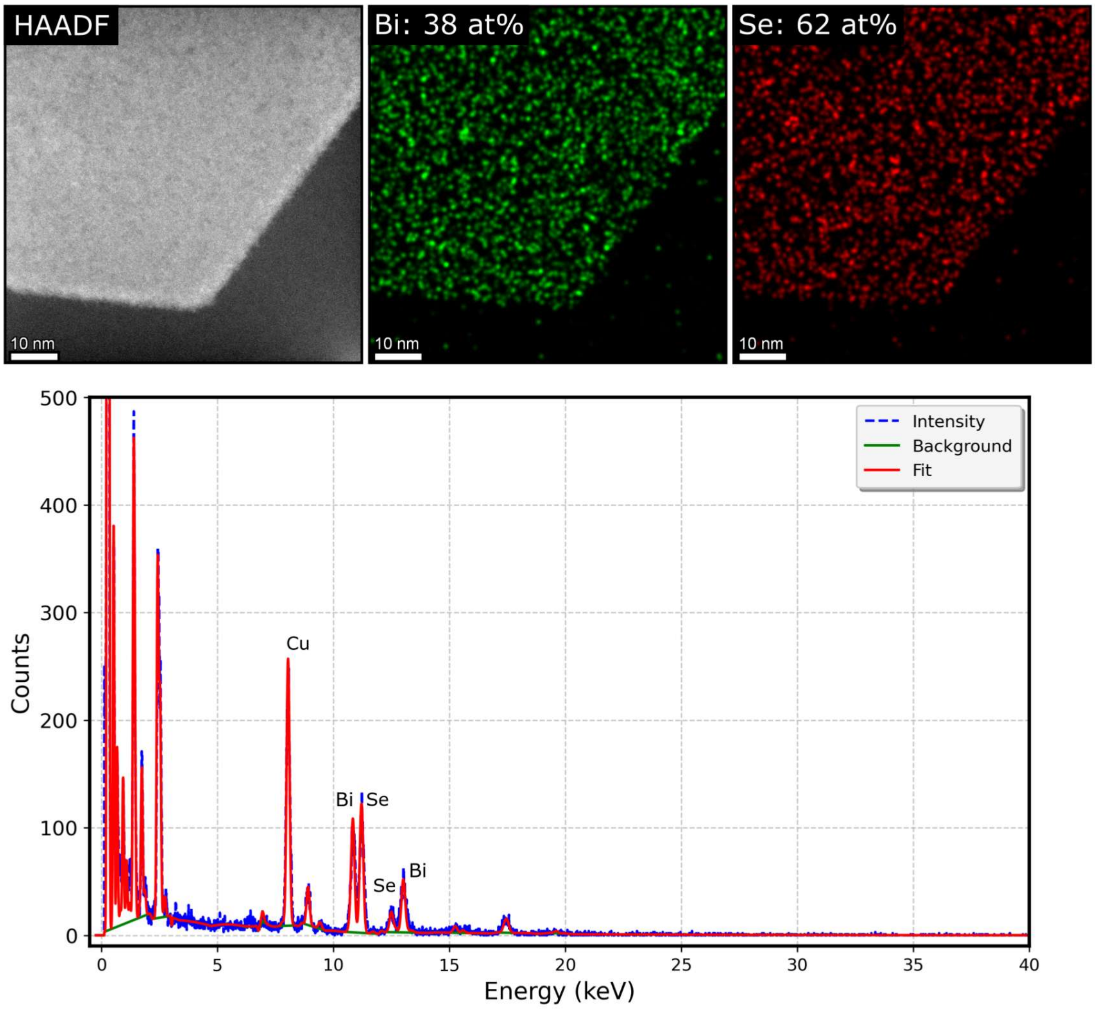

Fig. S23: **STEM-EDX measurement of a nanoplatelet.**
STEM-EDX maps showing the net intensity and the calculated Bi and Se atomic percentages (at%) (estimated with 3.0 at% error). The corresponding EDX spectrum is shown below the maps.

References

(1) Sun, Y.; Cheng, H.; Gao, S.; Liu, Q.; Sun, Z.; Xiao, C.; Wu, C.; Wei, S.; Xie, Y. Atomically Thick Bismuth Selenide Freestanding Single Layers Achieving Enhanced Thermoelectric Energy Harvesting. *J. Am. Chem. Soc.* **2012**, *134* (50), 20294–20297.

(2) Kung, H. H.; Goyal, A. P.; Maslov, D. L.; Wang, X.; Lee, A.; Kemper, A. F.; Cheong, S.-W.; Blumberg, G. Observation of Chiral Surface Excitons in a Topological Insulator Bi_2Se_3. *Proc. Natl. Acad. Sci. U. S. A.* **2019**, *116* (10), 4006–4011.

(3) Moes, J. R.; Vliem, J. F.; de Melo, P. M. M. C.; Wigmans, T. C.; Botello-Méndez, A. R.; Mendes, R. G.; van Brenk, E. F.; Swart, I.; Maisel Licerán, L.; Stoof, H. T. C.; et al. Characterization of the Edge States in Colloidal Bi_2Se_3 Platelets. *Nano Lett.* **2024**, *24* (17), 5110–5116.

(4) Schiettecatte, P.; Geiregat, P.; Hens, Z. Ultrafast Carrier Dynamics in Few-Layer Colloidal Molybdenum Disulfide Probed by Broadband Transient Absorption Spectroscopy. *J. Phys. Chem. C* **2019**, *123* (16), 10571–10577.

(5) Schiettecatte, P.; Hens, Z.; Geiregat, P. A Roadmap to Decipher Ultrafast Photophysics in Two-Dimensional Nanomaterials. *J. Chem. Phys.* **2023**, *158* (1), 014202.
